# Somatic genetic alterations in the development and progression in thyroid tumors of follicular cells

**DOI:** 10.1530/ETJ-25-0104

**Published:** 2025-10-29

**Authors:** Giulia Calafato, Floriana Jessica Di Paola, Antonio De Leo, Thais Maloberti, Sara Coluccelli, Laura Poppi, Andrea Repaci, Erica Solaroli, Stefania Damiani, Stefano Chillotti, Federico Chiarucci, Kerry Jane Rhoden, Dario de Biase, Giovanni Tallini

**Affiliations:** ^1^ Biobank of Research, IRCCS Azienda Ospedaliero-Universitaria di Bologna, Bologna, Italy; ^2^ Department of Medical and Surgical Sciences (DIMEC), University of Bologna, Bologna, Italy; ^3^ Solid Tumor Molecular Pathology Laboratory, IRCCS Azienda Ospedaliero-Universitaria di Bologna, Bologna, Italy; ^4^ Division of Endocrinology and Diabetes Prevention and Care, IRCCS Azienda Ospedaliero-Universitaria di Bologna, Bologna, Italy; ^5^ Endocrinology Unit, Ospedale Maggiore, Bologna, Italy; ^6^ Pathology Unit, IRCCS Azienda Ospedaliero-Universitaria di Bologna, Bologna, Italy; ^7^ Pathology Unit, Ospedale Maggiore, Bologna, Italy; ^8^ Department of Pharmacy and Biotechnology (FaBit), University of Bologna, Bologna, Italy

**Keywords:** anaplastic thyroid carcinoma, follicular thyroid carcinoma, molecular diagnostics, papillary thyroid carcinoma, pathways dysregulations, poorly differentiated thyroid carcinoma, thyroid cancer, thyroid genetic alterations, thyroid tumors

## Abstract

Thyroid cancer is the most prevalent endocrine malignancy. Distinct genetic alterations drive the development and progression of thyroid tumors of follicular cells with remarkable genotype–phenotype correlation. In most tumors of follicular cell origin, the primary molecular events are *RAS* or RAS-like (follicular-patterned tumors) and *BRAF* p.V600E or BRAF V600E-like (conventional papillary carcinomas) alterations. Progression of thyroid tumors to advanced and less-differentiated carcinomas requires additional oncogenic alterations, including *TP53* and *TERT* promoter mutation, and aberrant PI3K–PTEN–AKT signaling. Understanding the genetic landscape of thyroid carcinoma of follicular cells is essential to optimize clinical management and to identify molecular targets to treat cases with aggressive disease refractory to standard radioactive iodine therapy. What follows is a comprehensive and updated outline of the main somatic genetic and molecular alterations in thyroid carcinoma of follicular cells.

## Introduction

Thyroid cancer is the most prevalent endocrine malignancy, accounting for 4.1% of all newly diagnosed cancer cases globally, the 10th most common cancer worldwide (1, 2, 3). The widespread adoption of imaging techniques and the introduction of fine-needle aspiration (FNA) have greatly enhanced the ability to detect this malignancy. Indeed, the number of cases is expected to increase globally by 44.1% between 2019 and 2030 (4). However, this diagnostic progress has also raised concerns about the potential for overdiagnosis, where clinically insignificant cases are identified and treated unnecessarily (5). Thyroid cancer includes various histotypes that differ in frequency, mutational profile, clinical behavior and outcome (Table 1). While the overall 5-year survival rate for primary thyroid cancer is about 99%, it decreases significantly in advanced and less-differentiated tumors. Based on the 5th edition of the WHO classification scheme, tumors of follicular cells can be broadly divided into three groups (6). The first one includes three well-differentiated subtypes derived from follicular cells, such as papillary thyroid carcinoma (PTC), follicular thyroid carcinoma (FTC) and oncocytic carcinoma of the thyroid (OCA). The second group includes the rare and aggressive anaplastic (undifferentiated) thyroid carcinoma (ATC), which typically develops from well-differentiated thyroid carcinomas after accumulation of further genetic alterations. ATC has very poor outcome compared to both PTC and FTC that typically have a good prognosis and a 5-year survival rate close to 100% when localized to the thyroid gland (7, 8, 9). In addition, the current WHO classification of thyroid neoplasms recognizes a third group of tumors with an intermediate prognosis between that of well-differentiated PTC or FTC and undifferentiated anaplastic carcinomas: the high grade, non-anaplastic thyroid carcinomas (6). This group includes tumors that are poorly differentiated (poorly differentiated thyroid carcinoma, PDTC) and tumors that are well differentiated, but of high histologic grade (high-grade differentiated thyroid carcinoma, HGDTC). These high-grade non-anaplastic thyroid carcinomas of follicular cells have a 5-year survival of ∼50–70% (10, 11, 12). Medullary thyroid carcinoma (MTC), accounting for 3–5% of thyroid cancers, is not of follicular cell derivation, but is a neuroendocrine tumor originating from C-cells (parafollicular cells). Around 25% of MTC are inherited, in the context of multiple endocrine neoplasia (MEN) syndromes (13).

**Table 1:** Thyroid carcinoma: summary of histologic features, grading and molecular profile (WHO 5th edition).

	Conventional PTC	Infiltrative FVPTC	I-EFVPTC	FTC	OCA	HGDTC	PDTC	ATC	MTC
Precursor lesion	Unknown	Unknown	NIFTP (after acquiring invasion)	Follicular adenoma (after acquiring invasion)	Oncocytic adenoma (after acquiring invasion)	Progression from pre-existing tumors	Progression from pre-existing tumors with partial loss of follicular cell differentiation	Progression from pre-existing tumors with complete loss of follicular cell differentiation	C-cell hyperplasia
Architecture	Papillary	Follicular	Follicular	Follicular	Follicular, solid/trabecular	Papillary or follicular	Solid/trabecular/insular	Solid	Solid
Nuclei	PTC nuclear atypia (florid)	PTC nuclear atypia (florid)	PTC nuclear atypia (moderate)	Unremarkable	Enlarged, with prominent nucleoli	Alterations depend on tumor subtype	Small and round, may be convoluted, ‘rasin-like’	Pleomorphic	‘Salt and pepper’ chromatin
Mitoses	<5 mitoses/2 mm^2^	<5 mitoses/2 mm^2^	<5 mitoses/2 mm^2^	<5 mitoses/2 mm^2^	<5 mitoses/2 mm^2^	≥5 mitoses/2 mm^2^ or tumor necrosis	≥3 mitoses/2 mm^2^ or tumor necrosis	Numerous mitoses and necrosis	If ≥ 5 mitoses/2 mm^2^, Ki67 ≥ 5% or tumor necrosis MTC is high grade
Necrosis	No	No	No	No	No				
Invasive pattern	Infiltrative; spreads through lymphatics first	Infiltrative; spreads through lymphatics first	Invasion of tumor capsule/blood vessels; spreads to distant sites	Invasion of tumor capsule/blood vessels; spreads to distant sites	Invasion of tumor capsule/blood vessels; spreads to distant sites	Through lymphatics or blood vessels (depending on subtype)	Wide invasion of capsule/blood vessels, may spread to lymph nodes	Highly infiltrative, spreads through lymphatics and blood vessels	Infiltrative; spreads through lymphatics and blood vessels
Subtypes	Many, (13 for the WHO 5th ed.) depending on cell type/growth pattern	No	Minimally invasive Encapsulated angioinvasive Widely invasive	Minimally invasive Encapsulated angioinvasive Widely invasive	Minimally invasive Encapsulated angioinvasive Widely invasive	HGDTC-PTC, HGDTC-FTC, HGDTC-oncocytic, HGDTC other	No	Patterns: sarcomatoid, epithelioid, squamous cell carcinoma-like	Medullary microcarcinoma
Molecular profile	BRAF-V600E-like	BRAF-V600E-like	RAS-like	RAS-like	mtDNA mutations/extensive LOH	BRAF-V600E-like (more common) or RAS-like (less common), TERTp mutation	RAS-like (more common) or BRAF-V600E-like (less commmon), TERTp mutation	BRAF-V600E-like (high MAPK signaling), TERTp and *TP53* mutation	*RET* or *RAS* mutation

ATC, anaplastic thyroid carcinoma; FTC, follicular thyroid carcinoma; FVPTC, follicular variant papillary carcinoma; HGDTC, high-grade-differentiated thyroid carcinoma; I- EFVPTC, invasive encapsulated follicular variant papillary carcinoma; LOH, loss of heterozigosity; MTC, medullary thyroid carcinoma; mtDNA, mitochondrial DNA; NIFTP, non-invasive follicular thyroid neoplasm with papillary-like nuclear features; OCA, oncocytic carcinoma of the thyroid; PTC, papillary thyroid carcinoma; PDTC, poorly differentiated thyroid carcinoma; TERTp, TERT promoter.

Historically, total thyroidectomy, often combined with radioactive iodine therapy, has been the standard treatment for most thyroid cancer types of follicular cells derivation. Advances in next-generation sequencing and other high-throughput techniques have significantly enhanced our understanding of the molecular landscape of thyroid carcinoma, allowing a remarkable correlation between histologic phenotype and genotype. These technologies have been instrumental in uncovering genomic alterations that may serve as therapeutic targets for aggressive tumors unresponsive to conventional radioiodine therapy (2, 14, 15, 16, 17, 18).

This review provides a comprehensive and updated analysis of the genomic and molecular alterations driving thyroid cancer and of their correlation with histopathologic features. It investigates how these alterations drive thyroid cancer onset and progression and evaluates their biological significance, highlighting their potential to guide more effective treatment strategies to improve patient outcome.

## Early/driver molecular changes in the development of thyroid tumors

**Figure 1:**
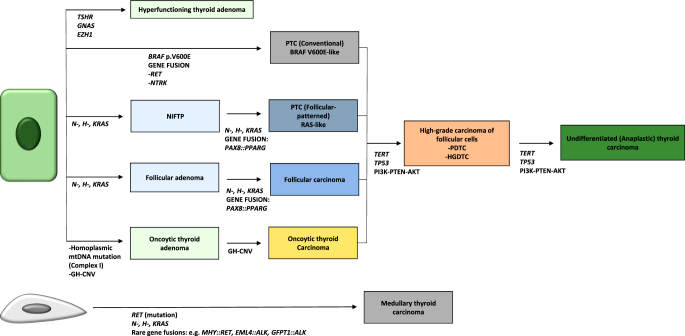
Somatic genetic alterations of thyroid tumors. PDTC, poorly differentiated thyroid carcinoma; HGDTC, high grade differentiated thyroid carcinoma; GH-CNV, genome haploidization-type DNA copy number variation. Modified from (19).

The main alterations of thyroid cancer of follicular cells fall into two broad categories: driver alterations that promote tumor development and secondary events that promote progression to high grade tumors and anaplastic carcinoma (Fig. 1, Tables 2 and 3) (2, 17, 18, 19, 20). The most frequent genetic variants linked to the development of thyroid cancer are found in proteins involved in the intracellular mitogen-activated protein kinase (MAPK) signaling pathway (21). Mutations or alterations that aberrantly activate the MAPK pathway leading to its dysregulation result in impaired expression of genes required for normal thyroid function, uncontrolled cellular growth and cancer development (2, 17, 18, 19, 20, 22). Mostly, dysregulation arises through mutually exclusive mutations in either *BRAF* (60–80%) or *RAS* (20–40%) oncogenes. A subset of thyroid cancers is initiated by gene rearrangements involving receptor tyrosine kinase (RTK) genes, such as *RET*, *ALK* and *NTRK* (5–10%), that also activate the MAPK pathway (Table 2) (2, 17, 18, 19, 20, 21). Overall, the clonal nature and mutual exclusivity of genetic alterations in *BRAF*, *RAS*, *RET*, and other key genes indicate that a single genetic change is the primary initiating event in most thyroid cancers, with impressive genotype–phenotype correlation. As shown by the work of The Cancer Genome Atlas (TCGA), follicular-patterned tumors are typically linked to *RAS* or RAS-like mutations. In contrast, conventional papillary carcinomas (those forming papillae) are predominantly associated with *BRAF* p.V600E or BRAF V600E-like mutations, including *RET* and *NTRK* rearrangements (21). Indeed, Veschi *et al.* demonstrated that introducing *BRAF* p.V600E mutations into human thyroid progenitor cells through CRISPR–Cas9 gene editing is sufficient to recreate PTC, while introducing the *NRAS* p.Q61R mutation successfully reproduces FTC (23). Other forms of tumors, notably oncocytic tumors, are driven by somatic genetic alterations that are not primarily related to either RAS-like or BRAF V600E-like molecular changes, such as mtDNA mutations and genome haploidization (20, 24, 25, 26, 27, 28, 29).

**Table 2:** Somatic genetic alterations of thyroid tumors: early/driver events.

Gene	Protein	Main genetic alterations	Main molecular pathway	Tumor type	Histologic features	References
*H-, K-, NRAS*	HRAS, KRAS and NRAS: G-proteins essential for the transmission of signals from the cell membrane through MAPK pathway	Mutations in the GTPase domain (codon 61), or in the GTP-binding domain (codons 12 and 13) lock the protein	MAPK	FTA^a^: 20–40%	Follicular-patterned tumors: FTA, FTC; if nuclear alterations of papillary carcinoma present: NIFTP, I-EFVPTC	(6, 12, 14, 18, 21, 30, 33, 34, 38, 52, 56)
				FTC^a^: 20–50%		
				PTC: 0–10%		
				HGDTC: 30%		
				PDTC: 50%		
				ATC: 10–50%		
*BRAF*	BRAF (along with ARAF and CRAF) is a serine/threonine kinase in the MAPK signaling pathway. Upon activation, BRAF phosphorylates and activates MEK, which subsequently activates ERK	Mutations, by far the most common is c.1799T > A (p.V600E) (95% of cases); other mutations that enhance *BRAF* activity occur in 599, 600, 601 and 469 codons (e.g., p.K601E, p.V600K, p.G469V), since the level of MAPK activation is lower, these mutations have a RAS-like signature	MAPK	PTC: 40–80%	Papillae (typically abundant), nuclear alterations of papillary carcinoma	(6, 12, 14, 18, 21, 38, 52)
				HGDTC: 50–60%		
				PDTC: 10%		
				ATC: 10–50%		
*RET*	RET^b^ is a transmembrane receptor tyrosine kinase regulating development of central and peripheral nervous systems; through a RET dimer–ligand–coreceptor complex binds ligands of the GDNF family	Fusion of RET tyrosine kinase domain with heterologous genes leading to intrachromosomal (most common: *CCDC6::RET* (previously RET/PTC1) and *NCOA4::RET* (previously RET/PTC3)) or interchromosomal (most common: *PRKAR1A::RET* (previously RET/PTC2)) rearrangements involving *RET* (10q11.2)	MAPK	PTC: 5–10%^c^	PTC with multinodular/lobulated growth, prominent intratumoral fibrosis, vascular invasion (lymphatics and/or blood vessels), solid/trabecular pattern or highly dense and glomeruloid papillae	(12, 14, 21, 38, 52, 71, 74, 75, 76, 77, 78, 79)
				PDTC, HGDTC, ATC: 0–5%		
*NTRK*	NTRK^b^ proteins are transmembrane receptor tyrosine kinases that regulate nerve cell differentiation; NTRK1 binds NGF, NTRK3 binds NT3	Fusion of NTRK1 and NTRK3 tyrosine kinase domain with heterologous genes leading to rearrangements involving *NTRK1* (1q21-22) (most common *NTRK1::TMP3* and *NTRK1::TPR*) and *NTRK3* (15q25) (most common *NTRK3::ETV6*)	MAPK	PTC: 5–10%^d^	Same histologic features of RET-rearranged cases	(12, 14, 21, 38, 52, 72, 73, 74, 76, 78, 79)
				HGDTC, ATC: 0–5%		
*ALK*	ALK^b^ is a transmembrane receptor tyrosine kinase regulating embryonal and neural development; ALK binds ALKAL proteins (FAM150, AUG)	Fusion of *ALK* (2p23.2-p23.1) with heterologous genes, most commonly: *ALK::STRN* (fusion of ALK exon 20 and STRK exon 3) and *ALK::EML2* (fusion of ALK exon 20 to EML4)	MAPK	PTC: 5%	Predominantly or entirely follicular growth pattern with small areas	(12, 14, 21, 38, 52, 73, 74, 76, 80, 83)
				PDTC, ATC: 0–5%	Of papillae growth; diffuse sclerosing papillary carcinoma; PDTC	
*TSHR*	TSHR is a member of the G-protein-coupled transmembrane receptor family	Mutations of *TSHR* (14q31.1) occur in regions involved in G-protein interaction leading to constitutive activation of TSHR, high cAMP levels and thyroid hormone production	cAMP	Hyperfunctioning thyroid adenoma^e^	Follicular patterned	(111, 112, 113)
*EIF1AX*	EIF1AX is a protein involved in protein translation initiation	Mutations of EIF1AX (Xp22.12) typically occur in exons 2, 5, and 6	Aberrant translation	FND, FTA^a^, FTC^a^: 0–5%	Follicular patterned	(12, 14, 21, 38, 49, 50, 51, 52)
				HGDTC: 0–5%		
				PDTC: 10%		
				ATC: 5–15%^f^		
*PPARG*	PPARG is a nuclear receptor protein regulating lipid uptake, adipogenesis and glucose metabolism. Fatty acids are physiological *PPARG* ligands	*PAX8::PPARG:* fusion of the coding sequence of *PAX8* (2q13) with the coding exons of *PPARG* (3p25); *CREB3L2::PPARG:* in-frame fusion of exons 1–2 of *CREB3L2* (7q34) with all coding exons of *PPARG* (3p25)	The chimeric oncoproteins (PPFP and CREB3L2:PPARG respectively) stimulate thyroid cell proliferation	FTA^a^5–20%	Follicular patterned	(12, 14, 21, 38, 44, 45, 48, 52)
				FTC^a^: 10–50%		
				PDTC, HGDTC, ATC: 0–5%		
*DICER1*	DICER1 is a ribonuclease involved in microRNA precursor processing	Somatic *DICER1* (14q32.13) mutations cluster in the RNase IIIb protein domain; germline mutations have no hot spots	microRNA deregulation, MAPK	FTA^a^, FTC^a^, PTC, PDTC, HGDTC: 1–2%	Follicular pattern and/or papillary architecture	(12, 85, 86, 87, 93, 94, 97)
mtDNA encoded oxidative phosphorylation (OXPHOS) genes; most mutations involve complex I	OXPHOS proteins are responsible for electron transport and the generation of a proton gradient across the mitochondrial inner membrane to drive ATP production	Accumulation of abundant homoplasmic mitochondria, near-total haploidization of the nuclear genome and mutations in genes encoding components of mitochondrial respiratory complex I	Reconfiguration of metabolic profile	OA and OCA: >80%	Oncocytic (adenoma, carcinoma)	(24, 25, 28, 29, 107)
GH-CNV: genome haploidization-type DNA copy number variation	NA (not applicable)	Genome haploidization at chromosomal level	NA (not applicable)	OA: 30–40%	Oncocytic (adenoma, carcinoma)	(26, 27, 28, 29, 106, 109, 110)
				OCA: >80%		
*APC* and *CTNNB1*	In the absence of Wnt ligand, APC protein, promotes β-catenin degradation; in the presence of Wnt ligand, β-catenin accumulates and translocates into the nucleus where promote the transcription of Wnt target genes	Germline or somatic *APC* loss of function mutations and somatic *CTNNB1* mutations (exon 3) stabilize the *β*-catenin protein, preventing its degradation and promoting its accumulation in the nucleus where induced the uncontrolled transcription of proliferation-related Wnt target genes	Wnt	CMCT: 100%	Uncertain histogenesis with endodermal (intestinal-like) differentiation and cribriform morular architecture	(116, 119, 121, 122)

^a^ Encapsulated follicular variant papillary thyroid carcinoma, with invasion or without invasion (NIFTP), have molecular alterations similar to follicular carcinoma and follicular adenoma, respectively.

^b^ RET, NTRK1, NTRK3, and ALK are not normally expressed in follicular thyroid cells; expression of their tyrosine kinase domain is driven by rearrangement with heterologous genes.

^c^ The prevalence of RET rearranged cases is higher in children and young patients, and in radiation-associated carcinomas.

^d^ The prevalence of NTRK rearrangements is variably reported between 0 and 5% for NTRK1 and NTRK3 in most series from non-radiation associated papillary carcinoma in adult patients; the prevalence is higher in children and young patients, and in radiation-associated papillary carcinoma.

^e^ Hyperfunctioning follicular adenomas and adenomatous nodules have mutations of the TSH gene receptor (*TSHR*) (about 50–80% of cases), of the *GNAS* gene (about 5% of cases), of the *EZH1* gene (about 20–30% of cases, nearly always in combination with *TSHR* or *GNAS* mutations, or with other alterations in cAMP pathway genes).

^f^ The combination of *EIF1AX* mutation with *RAS* mutations has been reported in aggressive thyroid carcinomas (PDTC, HGDTC, and ATC), even in the absence of other late/progression-associated events.

ALK, anaplastic lymphoma kinase; APC, adenomatous polyposis coli; ATC, anaplastic thyroid carcinoma; CMCT, cribriform morular thyroid carcinoma; EIF1AX, eukaryotic translation initiation factor 1A, X-linked; FTA, follicular thyroid adenoma; FND, follicular nodular disease; FTC, follicular thyroid carcinoma; HGDTC, differentiated high-grade thyroid carcinoma; MAPK, mitogen-activated protein kinase; MEN, multiple endocrine neoplasia; MTC, medullary thyroid carcinoma; NTRK1, neurotrophic tyrosine kinase receptor type 1; NTRK3, neurotrophic tyrosine kinase receptor type 3; OA, oncocytic adenoma; OCA, oncocytic carcinoma of the thyroid; PI3K, phosphatidylinositol 3-kinase; PDTC, poorly differentiated thyroid carcinoma; PPARG, peroxisomal proliferator-activated receptor gamma; PTC, papillary thyroid carcinoma; PTEN, phosphatase and tensin homolog; TC, thyroid cancer; TSHR, thyroid-stimulating hormone receptor.

**Table 3:** Somatic genetic alterations of aggressive thyroid tumors: late/progression-associated events^a^.

Genetic alterations^a^	Protein	Impaired molecular pathway/biological process	Tumor type	Co-mutated setting^b^	Putative mechanism involved	References
*PTEN-*loss of function mutations	PTEN blocks PI3K signaling through PIP3 dephosphorylation, inhibiting PIP3-dependent-AKT activation, ultimately reducing cell survival, growth and proliferation	PI3K–PTEN–AKT	FTC^c^: 0–10%	*RAS* mutations, less commonly *BRAF* p.V600E	Metastasis, cyclin D1-dependent cell cycle dysregulation, immunosuppressive microenvironment, cell cycle dysregulation, increased metabolism	(12, 14, 38, 52, 127, 133, 136, 138, 140, 143)
			HGDTC: 0–5%			
			PDTC: 10–20%			
			ATC: 10–15%			
*PIK3CA*-activating mutations	Upon growth factor receptor activation, PIK3 phosphorylates AKT, ultimately promoting cell growth and proliferation	PI3K–PTEN–AKT	HGDTC: 5%	*RAS* mutations*, BRAF* p.V600E	Epithelial mesenchymal transition	(12, 14, 38, 52, 132, 133, 144, 145)
			PDTC: 0–5%			
			ATC: 5–25%			
*TP53*-loss of function mutations	p53 is implicated in cell cycle control, DNA repair and stress-response apoptosis, thereby preventing uncontrolled cell proliferation	Cell cycle control, DNA repair system	HGDTC: 5%	*RAS* mutations*, BRAF* p.V600E, other driver alterations	Thyroid dedifferentiation, uncontrolled tumor growth, epithelial mesenchymal transition, MAPK, PIK-PTEN-AKT, genomic instability, DNA damage, metastases	(12, 14, 38, 52, 124, 126, 143, 155, 157)
			PDTC: 15–20%			
			ATC: 40–80%			
*TERT* promoter mutations	TERT belongs to the catalytic subunit of telomerase which add telomeres at the end of chromosomes, maintaining their integrity and genome stability	Chromosomal integrity, genome stability	FTC^c^: 10–35%	*RAS* mutations*, BRAF* p.V600E, other driver alterations	Aberrant TERT expression, leading to enhanced telomerase activity and increased telomere length	(11, 12, 14, 15, 37, 38, 52, 169, 170, 172)
			PTC: 5–15%			
			HGDTC: 60%			
			PDTC: 50%			
			ATC: 30–75%			
*SWI/SNF (ARID1A, ARID1B, ARID2, SMARCB1* or *PBRM1)* mutations	SWI/SNF (subfamily of ATP-dependent chromatin remodeling complexes) mobilize nucleosomes and remodel chromatin, regulating transcription of target genes	Chromatin-remodeling activity, differentiation-associated gene expression	HGDTC, PDTC: 10%	*RAS* mutations*, BRAF* p.V600E	De-differentiation, stem-cell-like properties	(12, 14, 38, 52, 176, 177, 178)
			ATC: 15–35%			
*NF2*-loss of function mutations	MERLIN (encoded by NF2) prevents the YAP-dependent activation of TEAD-mediated transcription of growth-associated genes	Hippo pathway (YAP/TAZ-TEAD)	HGDTC, PDTC: 0–5%	*RAS* mutations*, BRAF* p.V600E	YAP/TAZ-TEAD driven RAS transcription	(12, 14, 38, 52, 180)
			ATC: 10%			

^a^ Alterations typically overlap with early driver events (cfr. Table 1).

^b^ Late/progression-associated events, particularly TP53 and TERT promoter mutation, may also overlap with driver alterations of oncocytic tumors (mtDNA mutations and genome haploidization).

^c^ Encapsulated follicular variant papillary thyroid carcinoma with invasion of tumor capsule or blood vessels has molecular alterations similar to follicular carcinoma.

AKT, Ak strain transforming, AKT serine/threonine kinase 1; ATC, anaplastic thyroid carcinoma; FTC, follicular thyroid carcinoma; HGDTC, differentiated high-grade thyroid carcinoma; MAPK, mitogen-activated protein kinase; MERLIN, moesin-, ezrin-, radixin-like protein; NF-kB, nuclear factor kappa-light-chain-enhancer of activated B cells; P53, tumor protein P53; PDTC, poorly differentiated thyroid carcinoma; PI3K, phosphoinositide 3-kinase; PTC, papillary thyroid carcinoma; PTEN, phosphatase and tensin homolog; SWI/SNF, switch/sucrose non-fermentable; TERT, telomerase reverse transcriptase; TAZ, transcriptional coactivator with PDZ-binding motif; TEAD, transcriptional enhancer factor TEF-1 also known as TEA domain family member 1; YAP, yes-associated protein.

### *H-*, *K-*, *NRAS* and RAS-like alterations

#### H-, K-, and NRAS

RAS genes, *HRAS*, *KRAS*, and *NRAS*, encode a family of G proteins that reside on the inner side of the cytoplasmic membrane, where they play a key role in transmitting intracellular signals to the nucleus. RAS proteins function as key effector molecules in MAPK signaling and are commonly activated in a variety of human cancers, contributing to oncogenesis (30). *RAS* mutations or similar molecular alterations (RAS-like) are 'early/driver' events for follicular-patterned tumors. These *RAS* or RAS-like mutated tumors exhibit a consistent molecular profile, low MAPK signaling (due to negative feedback from ERK to RAF monomers), a high differentiation score, and are malignant only if there is invasion into the tumor capsule or blood vessels (2, 18, 19, 20, 21).

RAS-encoded G-proteins, known as p21RAS GTPases, are inactive when bound to guanosine diphosphate (GDP) and active when bound to guanosine triphosphate (GTP). A group of proteins, including guanine nucleotide exchange factors (GEFs) and GTPase-activating proteins (GAPs), facilitate the activation of p21RAS by promoting the exchange of GDP for GTP. Point mutations in the GTP-binding domain (codons 12 and 13) or the GTPase domain (codon 61) lead to amino acid substitutions that impair GTPase activity, causing p21RAS to remain in its active form, driving tumorigenesis. In human cancer, more than 90% of RAS mutations are found in codons 12, 13, or 61 (31, 32).

In thyroid tumors, mutations most commonly involve *NRAS* (at codon 61) found in 25–80% of FTC and 15–65% of follicular thyroid adenoma (FTA). *NRAS* mutations are followed – in order – by those of *HRAS* (5–20% in FTC and 5–35% in FTA) and *KRAS* mutations (<5% in both FTC and FTA) (33, 34). RAS-mutated thyroid tumors are typically follicular-patterned. RAS mutations also characterize those encapsulated follicular-patterned tumors that have nuclear alterations of papillary carcinoma: invasive encapsulated follicular variant of papillary thyroid carcinoma (I-EFVPTC), an invasive tumor equivalent to FTC, and non-invasive follicular thyroid neoplasm with papillary-like nuclear features (NIFTP), a non-invasive tumor equivalent to FTA (35, 36). The prevalence of *RAS* mutations in I-EFVPTC and NIFTP is similar to that of FTC and FTA, respectively (33, 34, 35, 36).

*RAS* mutations are found in aggressive, high-grade tumors that arise from the progression of these follicular-patterned neoplasms; they are reported in 20–50% of high-grade non-anaplastic carcinomas and in 10–50% of anaplastic carcinomas (Fig. 1 and Table 2) (12, 14, 19, 20, 21, 37, 38). Importantly, *RAS* mutations are also found in 5–10% of hyperplastic-appearing nodules in benign follicular nodular disease of the thyroid gland (FND, previously multinodular goiter) (6, 34). Thus, although the presence of *RAS* mutations suggests that a thyroid nodule is more likely a neoplasm rather than a hyperplastic nodule, these mutations do not necessarily correlate with malignancy (19).

Somatic *RAS* mutations occur in approximately 20% of MTC. In the latter, *RAS* mutations are mutually exclusive with somatic *RET* mutations and rare in patients affected by multiple endocrine neoplasia type 2 (MEN2) due to germline *RET* mutation. Notably, the mutation frequencies differ from those seen in follicular cell-derived tumors, with *HRAS* being the most frequently mutated gene (∼25% of MTC), followed by *KRAS* (∼15% of MTC), while *NRAS* mutations are very uncommon (less than 5%) (39, 40, 41). Overall, *H-*, *K-*, and *NRAS* mutations represent the most frequent genetic alterations found in thyroid FNA after *BRAF* mutations (42). Thyroid carcinomas with *RAS* mutations are prone to vascular invasion but retain the ability to respond to radioactive iodine.

Currently, there are no drugs targeting *RAS*-mutated forms of thyroid carcinoma (2, 17, 37, 43).

The follicular-patterned neoplasms described above can also be initiated by events genetically unrelated to MAPK signaling but that generate a RAS-like signature and histologic features.

#### PAX8::PPARG

The PAX8–PPARG fusion oncoprotein is the product of a rearrangement involving the thyroid transcription factor PAX8 that drives the expression of PPARG, a nuclear receptor with a role in adipocyte differentiation (44, 45, 46). PAX8::PPARG has been reported in up to ∼35% of FTC, and in I-FVPTC, and in some FTA and NIFTP (21, 44, 45, 46). Other genes may be fused with *PPARG*, such as *CREB3L2* (47). The conditional expression of PAX8–PPARG together with *PTEN* loss in transgenic mice induces thyroid tumors and enhances tumor levels of phospho-AKT (48).

#### EIF1AX

*EIF1AX* mutations impair protein translation initiation, causing aberrant translation (21, 49, 50, 51). They occur in a small subset of a variety of follicular-patterned lesions, including hyperplastic-appearing nodules in FND, FTA, FTC, NIFTP and I-EFVPTC (21, 49, 50, 51). While the cancer initiation potential of *EIF1AX* mutations is lower compared to that of other driver events, as they are also found in benign nodules, including FND, these mutations can promote thyroid cancer progression when combined with other MAPK-activating events, in particular, *RAS* mutations (14, 52).

### *BRAF* p.V600E and BRAF V600E – like alterations

#### *BRAF* p.V600E

**Figure 2:**
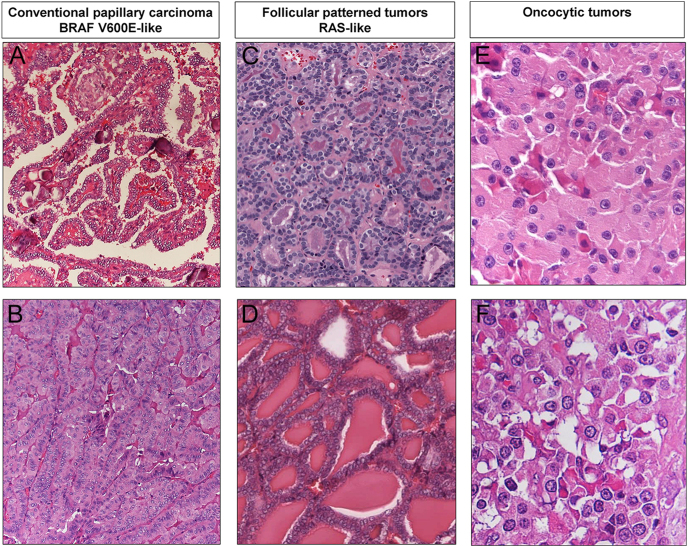
(A) Classic papillary carcinoma (100X); (B) tall cell subtype of papillary carcinoma: conventional papillary thyroid carcinomas have BRAF V600E-like molecular profile (100X); (C) follicular carcinoma (100X); (D) invasive encapsulated follicular variant papillary carcinoma: follicular-patterned tumors have RAS-like molecular profile (100X); (E) oncocytic adenoma (200X); (F) poorly differentiated oncocytic carcinoma: oncocytic tumors have a molecular profile characterized by mtDNA mutations and genome haploidization (200X).

*BRAF* is located at 7q34 and a single nucleotide substitution in exon 15, in particular, a transversion at position 1,799 (c.1799T > A) causes replacement of the hydrophobic valine with glutamic acid at codon 600, leading to the persistent activation of MEK signaling promoting tumorigenesis (53, 54). *BRAF* p.V600E or equivalent molecular alterations (BRAF V600E-like signature) are the 'early/driver' events in the development of conventional papillary carcinoma (Figs 1 and 2 and Table 2). Following the current 5th edition of the WHO classification scheme, ‘conventional’ papillary carcinoma refers to all papillary carcinoma subtypes with the exception of the follicular variant papillary carcinoma with invasion of tumor capsule or blood vessels (I-EFVPTC), that is a RAS/RAS-like mutated tumor, and is not considered anymore a papillary carcinoma subtype (6). BRAF V600E-like tumors exhibit a low differentiation score together with a heterogeneous molecular profile, characterized by elevated MAPK signaling due to the absence of negative feedback from ERK to RAF monomers (2, 19, 20, 21, 55).

*BRAF* p.V600E is the most common *BRAF* mutation found in human cancers (54). It represents a hallmark driver alteration in PTC with the well-known alterations of nuclear morphology – nuclear clearing, irregular contours of the nuclear membrane, grooves, and pseudoinclusions – that characterize this tumor type (6, 56). ‘Conventional’ papillary carcinomas are characterized by infiltrative growth and the formation of papillae. However, in some cases, they may exhibit features that are less typical, such as infiltrative follicular or solid/trabecular growth patterns. These variations contribute to the diverse subtypes of papillary carcinoma, each displaying unique histological traits (6). *BRAF* p.V600E is present in 40–80% of PTC cases but is essentially absent in follicular-patterned tumors (FTA, FTC, NIFTP, and I-EFVPTC) (6, 56). *BRAF* p.V600E is also detected in aggressive, high-grade tumors arising from the progression of conventional papillary carcinoma, with reported frequencies ranging from 10 to 50–60% for high-grade non-anaplastic carcinomas (HGDTC and PDTC) and from 10 to 50% for anaplastic carcinomas (Fig. 1 and Table 2) (6, 12, 19, 37, 38).

The prognostic significance of the *BRAF* p.V600E mutation is controversial, some studies having shown an association with poor outcomes (57, 58), others not (59, 60, 61). *BRAF* p.V600E has been linked to lymph node and distant metastases, advanced disease stage, decreased expression of genes involved in thyroid hormone biosynthesis, reduced responsiveness to radioiodine therapy, and an increased risk of recurrence (62, 63, 64, 65).

However, the overall prognostic relevance of the mutation is very limited when other factors, such as tumor stage and papillary carcinoma subtype, are considered (2, 9, 59, 66).

Mitsutake *et al.* demonstrated that in thyroid PC33L cells, *BRAF* p.V600E expression provides only a modest growth advantage, as it simultaneously activates DNA synthesis and apoptosis. However, unlike *RET* rearrangements, *BRAF* p.V600E may promote the acquisition of secondary genetic alterations by inducing genomic instability, which could contribute to its more aggressive behavior (22).

Notably, advanced *BRAF* p.V600E-mutated carcinomas, including anaplastic and iodide-refractory non-anaplastic carcinomas, can be treated with molecularly targeted therapies, such as dabrafenib (BRAF inhibitor) and trametinib (MAPK inhibitor). Therefore, identifying this mutation is not only important for diagnosis but also for predicting the tumor’s potential response to targeted molecular treatments (2, 17, 37, 43).

Although *BRAF* p.V600E mutations account for over 95% of all *BRAF* mutations identified in thyroid carcinoma (54), other non-V600E *BRAF* mutations, including substitutions or small insertion–deletions within the activation loop (residues 596–601) or in residues of the DFG (Asp–Phe–Gly) motif (residues 594–596), can also occur (67, 68, 69). Mutations in codons 599–601 (e.g. p.V600E, p.K601E, and p.V600K) and other substitutions that also enhance BRAF activity leading to constitutive activation of the protein have a more limited oncogenic potential (68, 70), as do small insertions–deletions (e.g. *BRAF* p.V600delinsNM, *BRAF* p.V600_K601delinsQ, and *BRAF* p.K601_S605delinsN). Unlike *BRAF* p.V600E-mutated tumors, which are typically conventional papillary carcinomas, tumors with non-V600E *BRAF* mutations tend to have a follicular pattern and are often histologically classified as follicular adenoma or carcinoma, I-EFVPTC, or NIFTP (67, 69).

#### Tyrosine kinase fusion and related rearrangements

An alternative mechanism through which thyroid cells can activate the MAPK kinase is through the rearrangement of tyrosine kinase genes. In human thyroid tissue, more than 20 receptor proteins with tyrosine kinase activity have been identified, several of which have been associated with thyroid cancer. The most significant of these includes *RET*. About 5–10% of adult PTC are driven by gene rearrangements that lead to the reactivation of the kinase domain of *RET* proto-oncogene (Fig. 1 and Table 2). The latter is localized on chromosome 10 (10q11.2) and has 21 exons encoding a transmembrane tyrosine-kinase receptor (71). These *RET* fusions are mutually exclusive with point mutations in *BRAF* or *RAS*, and with *BRAF* fusions.

The tyrosine kinase domain of *RET* undergoes rearrangement with various partner genes expressed in thyroid follicular cells, resulting in several aberrant fusion transcripts with upregulation and aberrant expression of RET tyrosine kinase in neoplastic follicular cells. The most common *RET* fusions are *CCDC6::RET* (formerly RET/PTC1) and *NCOA4::RET* (formerly RET/PTC3), which together account for over 90% of RET-rearranged cases (71). *RET* rearrangements are more common in pediatric and young adult populations and in patients with radiation-induced papillary carcinoma (72, 73, 74). *RET*-rearranged papillary carcinomas often display crowded papillae with few follicular structures, and some have the features of the diffuse sclerosing subtype of papillary carcinoma (73, 75, 76, 77). *RET*-rearranged carcinomas are typically aggressive at presentation, but generally have a favorable prognosis, as they tend to respond to radioactive iodine therapy (2, 17, 19, 20, 37, 43, 78).

A small subset of PTC is driven by fusions involving RTKs other than *RET*. These rearrangements typically involve *NTRK1*, *NTRK3*, or *ALK*, with various fusion partners (72, 73, 74, 79, 80), and have been shown to induce thyroid tumors *in vivo* (Fig. 1 and Table 2) (81, 82).

*NTRK* genes, particularly *NTRK1* and *NTRK3*, are frequently rearranged in thyroid follicular cell tumors, with mechanisms similar to those for *RET*. These rearrangements involve the fusion of the tyrosine kinase domain of *NTRK* with partner genes, leading to the expression of aberrant fusion transcripts (72, 73, 74, 78, 79, 80).

*NTRK*-rearranged tumors resemble BRAF V600E-like conventional papillary carcinomas. Tumors often present with fused papillae and convoluted glomeruloid structures (73, 75). *NTRK*-rearranged tumors predominantly occur in children and young adults (19, 20, 72, 73, 74, 78) and, like *RET*-rearranged tumors, can present aggressively. However, the prognosis is not necessarily poor, as they too usually respond to radioactive iodine treatment (73, 74, 78).

*ALK* is the third major tyrosine kinase gene commonly rearranged in thyroid tumors. *ALK*-rearranged carcinomas are less frequent than those with *RET* or *NTRK* rearrangements, and typically involve fusion with partner genes, such as *STRN* (encoding striatin) or *EML4* (also involved in lung adenocarcinoma) (75, 76, 80, 83). While most cases are classified as papillary carcinoma, the majority exhibit follicular growth pattern. Some *ALK*-rearranged carcinomas display the features of diffuse sclerosing papillary carcinoma, others have poorly differentiated morphology (73, 75, 76, 80, 83). *ALK*-rearranged tumors have been reported in individuals exposed to high doses of radiation (84). Genes less commonly rearranged in papillary carcinoma with BRAF V600E-like signature include *BRAF*, *MET* and *ROS1* (e.g., *CUL1-BRAF, MKRN1-BRAF, SND1-BRAF, TTYH3-BRAF CCDC30-ROS1 EML4-MET,* and *TFG-MET*) (73, 74, 75).

Overall, thyroid carcinomas with tyrosine kinase gene rearrangements share common clinicopathologic features. Their prevalence is higher in pediatric patients and young adults, and after radiation exposure. Presentation is often aggressive, with pT3 or pT4 disease, lymph node metastases and sometimes distant metastases. Histologically, they show multinodular and/or lobulated growth, prominent intratumoral fibrosis (confluent or arborizing), lymphovascular invasion, solid/trabecular or papillary patterns, with papillae that are highly dense and glomeruloid (19, 20, 73, 74, 75, 78).

The prevalence of tyrosine kinase rearrangements in conventional papillary carcinoma is lower compared to that of other driver mutations, such as *BRAF* p.V600E and *RAS.* Rearrangements are uncommon in advanced tumors, but they have been reported in up to 5–25% of aggressive, high-grade non-anaplastic tumors and in up to 5–10% of anaplastic carcinomas (14, 19, 37, 52). Aggressive radioiodine-resistant carcinomas with tyrosine kinase gene fusion can now be treated with specific molecularly targeted drugs, such as pralsetinib or selpercatinib for *RET*-rearranged tumors, larotrectinib for *NTRK*-rearranged tumors, and entrectinib for *ALK*- and *ROS1*-rearranged tumors, and testing for tyrosine kinase gene rearrangement is often requested to pathology laboratories (2, 75).

### Additional alterations

#### DICER1

A small subset, approximately 1–2%, of thyroid nodules harbor early-driver mutations in the *DICER1* gene, most of these nodules are benign, but some are clinically and histologically malignant (Table 2) (85). *DICER1* encodes a ribonuclease involved in processing microRNA precursors, and somatic mutations in the gene often cluster to the RNase IIIb protein domain. Mutations are mutually exclusive with canonical RAS-like or BRAF V600E-like drivers, suggesting a functional effect that disrupts microRNA processing and potentially activates MAPK signaling (86, 87). Indeed, transcriptomic analysis has shown that *DICER1*-mutated tumors have RAS-like molecular profile (88, 89). *DICER1* mutations typically occur in well-differentiated follicular-patterned tumors (FTA, FTC, NIFTP, and I-EFVPTC) (85, 90, 91, 92, 93, 94, 95, 96, 97, 98). Recent studies have demonstrated that a dominant macrofollicular growth pattern and areas with pale-stained involutional changes are two major histological features of follicular tumors harboring *DICER1* mutations (94, 99). *DICER1* mutations have also been reported in a few high-grade non-anaplastic carcinomas – including poorly differentiated thyroid carcinoma – and in rare anaplastic carcinomas (93, 95). *DICER1* mutations also characterize thyroblastoma, a very rare embryonal thyroid tumor (100, 101).

In patients with *DICER1* syndrome, characterized by germline *DICER1* mutation, somatic *DICER1* mutations in thyroid nodules represent a second hit. Somatic mutations occur at hotspot codons within the RNase IIIb regulatory domain, whereas germline variants tend to be truncating and are found throughout the entire gene. In general, while hotspot mutations are typically somatic, truncating mutations may be either germline or somatic (85, 97, 98). *DICER1* syndrome is characterized by both benign and malignant thyroid lesions, in addition to a wide range of neoplasms, including blastoma-type tumors (e.g., pleuropulmonary blastoma and Wilms tumor) and Sertoli–Leydig cell tumor, which develop in the pediatric age (85, 102, 103). Interestingly, *DICER1* mutations in thyroblastoma are somatic, not germline; thus, thyroblastoma is not currently recognized as a feature of *DICER1* syndrome (100, 101). In *DICER1*-mutated thyroid nodules, benign or malignant, diagnosed with nodular goiter, the prevalence of a coexisting germline mutation indicative of *DICER1* syndrome may be as high as 13% (104). Thus, even if the majority of *DICER1* mutations in thyroid tumors are somatic, germline testing should be performed to identify patients with *DICER1* syndrome (97, 98).

#### Oncocytic tumors of thyroid follicular cells

Mitochondrial DNA (mtDNA) mutations alongside significant genome haploidization-type DNA copy number variation (GH-CNV) represents an “early/driver” event for the development of oncocytic tumors (OCA, previously known as Hürthle cell carcinoma) (Fig. 1 and Table 2). This genetic pathway is distinctive of oncocytic tumors. Indeed, BRAF V600E-like or RAS-like mutations that characterize other follicular cell tumors are uncommon in oncocytic tumors (24, 25, 26, 27, 28, 29, 33). In two large series, *H-*, *K-*, and *NRAS* mutations were identified in less than 10% of carcinomas and *BRAF* p.V600E in less than 5% (24, 25). On the other hand, late/progression-associated genetic alterations are common in advanced tumors, particularly *TERT* promoter mutations found in up to approximately 60% of cases (52). The mtDNA mutations range from point substitutions to small insertions or deletions that can cause frameshifts or premature stop codons and large-scale deletions (29, 105, 106, 107). Several studies have revealed that the biochemical, metabolic, and phenotypic alterations of oncocytic tumors, such as the accumulation of mitochondria, are driven by homoplasmic mtDNA mutations in genes that code for subunits of the five multimeric complexes of the inner mitochondrial membrane, which are essential for the oxidative phosphorylation (OXPHOS) system. When these subunits are missing or defective due to mtDNA mutations, the assembly of the multimeric complex is disrupted, impairing OXPHOS and leading to a compensatory buildup of mitochondria (24, 25). In oncocytic thyroid tumors, and in those from other organs, more than 50% of mutations in mitochondrial genes are homoplasmic, with over 70% of these affecting MT-ND genes encoding subunits of Complex I (NADH coenzyme Q reductase) (Fig. 1 and Table 2) (24, 25, 28, 29). While mitochondrial gene mutations affecting OXPHOS function are responsible for the oncocytic phenotype and are also present in benign oncocytic lesions (25), their role in tumor development remains complex and not fully understood. Although both neoplastic and non-neoplastic oncocytic lesions harbor homoplasmic mtDNA mutations, oncocytic tumors in the thyroid and other organs are marked by significant DNA copy number variation, including widespread loss of heterozygosity at the chromosomal level (20, 26, 27). Although chromosomal DNA loss is extensive, some chromosomes are consistently (chromosome 7) or typically (chromosomes 5, 12, and 20) retained reflecting the presence of crucial imprinted genes in these chromosomes (26, 106). This near-complete haploidization of the genome may be followed by chromosomal endoreduplication, resulting in a “pseudodiploid” copy number-neutral uniparental disomy pattern across much of the genome (20, 26, 27). While mtDNA mutations are responsible for the oncocytic phenotype (25), the loss of chromosomal DNA has been linked to tumor development. Haploidization-type DNA copy-number changes are more commonly observed in oncocytic carcinomas than in oncocytic adenomas and are rare in hyperplastic oncocytic nodules (20, 28, 29, 108, 109). Indeed, aneuploidy detected by flow cytometry analysis of DNA content has long been linked to malignancy in oncocytic tumors (109, 110).

#### Other additional alterations

Other alterations include *TSHR*, *GNAS*, and *EZH1* mutations in hyperfunctioning thyroid adenomas and *wnt* pathway dysregulation in cribriform morular carcinoma (Table 2).

Hyperfunctioning thyroid adenomas develop due to alterations that promote thyroid hormone synthesis, secretion, and follicular cell proliferation. Activating mutations in the *TSHR* gene are present in up to 70% of hyperfunctioning thyroid nodules, while mutations in *GNAS* are found in a smaller subset (111, 112, 113). *TSHR*- and *GNAS*-mutated lesions are follicular-patterned and/or with a papillary architecture, and typically benign; however, the same mutations have also been reported in a few follicular carcinomas (18, 52). *TSHR* and *GNAS* mutations activate adenylyl cyclase, increasing intracellular cyclic AMP (cAMP) levels, thus leading to unregulated stimulation of thyroid follicular cell function and proliferation (114). *EZH1* mutations are associated with increased trimethylation of histone H3 and enhanced proliferation of follicular cells. *EZH1* mutations are typically found in combination with *TSHR* or *GNAS* mutation and occur in approximately 30% hyperfunctioning thyroid adenomas (115).

Dysregulated *wnt* activation is the defining feature of the rare cribriform morular carcinoma. The tumor develops in a sporadic form (116), or as an extraintestinal component of familial adenomatous polyposis syndrome (117), of which it may be the initial clinical manifestation (118). It is almost completely restricted to the female sex and it is currently considered of uncertain histogenesis (6), displaying a peculiar endodermal (intestinal-like) phenotype (119, 120).

*Wnt* dysregulation in thyroid tumors in patients with familial adenomatous polyposis is due to germline loss of function of *APC* (typically due to exon 15 mutations), typically followed by a somatic *APC* mutation as a second hit (121). In sporadic cribriform morular tumors, a combination of phenotypically equivalent somatic alterations in genes that activate *wnt* are observed, such as mutations in exon 3 of the *β*-catenin gene *(CTNNB1)* that stabilize the protein (122), *APC*, and/or *AXIN1* (119).

## Somatic genetic alterations of aggressive thyroid carcinoma: late/progression-associated events

**Figure 3:**
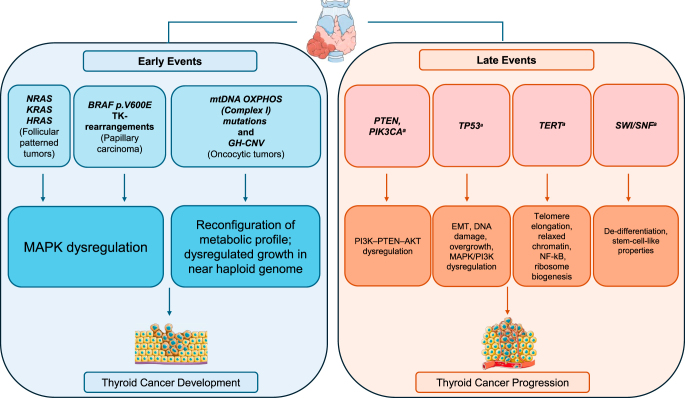
Somatic genetic alterations in thyroid tumors of follicular cells: early/driver- and late/progression-associated events. ^a^Co-mutation with early driver events. GH-CNV, genome haploidization-type DNA copy number variation; EMT, epithelial mesenchymal transition; TK, tyrosine kinase.

Understanding the genetic and biological mechanisms that underline thyroid tumor development and progression is an unavoidable step to improve the clinical management of patients with thyroid tumors, particularly of those with aggressive and advanced disease (17, 18, 43, 123). As previously discussed, clonal and mutually exclusive early/driver molecular alterations represent the initiating events in the development of follicular cell tumors, ultimately triggering a mutation-specific pattern of MAPK overexpression in follicular-patterned tumors and in papillary carcinomas (21). As in the case of other neoplasms, additional alterations are required for these well differentiated tumors to progress: secondary molecular mechanisms other than MAPK signaling underpin the transition to aggressive and less differentiated phenotypes (Figs 1 and 3). Indeed, oncogenic alterations in addition to the primary BRAF V600E-like, RAS-like or other less frequent drivers have been consistently identified in these aggressive tumors (14, 18, 19, 20, 37, 52, 124). In particular, in tumors where poorly or undifferentiated areas are associated with a well-differentiated component, ‘Early/driver’ alterations are identified in both areas, while ‘Late/progression’ changes are restricted to the less differentiated portions of the tumor (18, 125, 126). In the past decade, massive use of whole-genome and RNA-sequencing has revealed how specific combinations of primary driver and secondary changes acquired later during progression correlate with thyroid tumor aggressiveness and lack of response to radioiodine treatment (14, 18, 19, 20, 37, 52, 124, 127, 128). More recently, transcriptomics may provide additional insights into the mechanisms that underlie cancer progression and metastasis (129, 130). The main alterations involved in the progression of tumors of follicular cells include *PTEN*- or *PIK3CA*-driven aberrant PI3K–PTEN–AKT signaling, cell cycle dysregulation due to *TP53* loss-of-function, telomerase dysregulation caused by *TERT* promoter mutations, and SWI–SNF-dependent chromatin reconfiguration (Figs 1 and 3, and Table 3) (14, 18, 19, 20, 37, 52, 124).

### *PTEN* and *PIK3CA* mutation

The PI3K–PTEN–AKT pathway is physiologically involved in essential cellular processes, including protein synthesis, angiogenesis, metabolism, proliferation and survival. Pathologic overactivity of the pathway, driven by mutation of one of its effectors, contributes to uncontrolled growth, increased cell metabolism, invasion and metastasis. These processes are primarily caused by mRNA translation boosting and, secondarily, by feedback-positive-dependent overactivated MAPK signaling (131). In thyroid cancer, PI3K–PTEN–AKT dysregulation is mostly due to *PTEN* loss-of-function or to activating *PIK3CA* mutations, typically found in advanced and aggressive thyroid tumors (Figs 1 and 3 and Table 3) (14, 37, 52, 132, 133, 134). MicroRNA-dependent PI3K–PTEN–AKT dysregulation may be an additional mechanism in the progression of thyroid carcinoma (135). There is a specific pattern of PI3K–PTEN–AKT alteration with regards to MAPK-activating drivers: oncogenic *PIK3CA* mutations usually co-occur in *BRAF* p.V600E-mutated tumors, while *PTEN* mutation is more common in *RAS*-mutated ones (14, 134).

Genetically modified mouse models are useful to understand the mechanistic relationship between co-mutation and tumor progression with the aim of identifying specific molecular targets.

Several studies have reported how the combination of *PTEN* inactivation with either *KRAS* p.G12D or *HRAS* p.G12V causes cyclin D1-dependent cell cycle dysregulation, enhanced MAPK activation and an immunosuppressive tumor microenvironment, promoting metastatic spread and progression of follicular carcinoma (136, 137, 138). Transgenic mice models show that *PIK3CA (*p.H1047R*)* and *BRAF* p.V600E co-mutation induce epithelial–mesenchymal transition (EMT) in the tumor, which in turn is associated with decreased mouse survival and transition from well-differentiated thyroid tumors to anaplastic carcinoma (139). Although co-mutation of *BRAF* p.V600E and *PTEN* is unusual, in mice models, this co-mutation induces enhanced proliferation and progression of well-differentiated thyroid carcinoma to poorly differentiated and anaplastic forms (139, 140, 141, 142).

*PTEN* and *TP53* co-mutation, typical of a subset of anaplastic carcinomas, has been proven to dysregulate cell cycle and metabolism, thus promoting anaplastic transformation in mouse models (143).

Several studies have explored the PI3K–PTEN–AKT pathway as target for molecular therapy: double mutant *BRAF* p.V600E and *PIK3CA* thyroid carcinoma cells lines and xenograft models have heightened sensitivity to combination therapy with simultaneous MAPK and PI3K–PTEN-AKT pharmacological inhibition (144, 145). However, due to high related toxicity, dual targeting of MAPK and PI3K–PTEN–AKT pathways is difficult to translate into clinical practice (146).

### *TP53* loss-of-function

*TP53* gene encodes for the p53 protein, a tumor suppressor implicated in several biological processes as cell cycle control, DNA repair and stress-response apoptosis, thereby preventing uncontrolled cell proliferation (147). As a result, inactivation of *TP53* is common, with mutations occurring in more than 50% of human tumors, typically as a late event combined with earlier oncogenic changes (148, 149).

In thyroid carcinomas, *TP53* loss-of-function is associated with progression of differentiated carcinomas to anaplastic tumors (Figs 1 and 3 and Table 3). Indeed, the prevalence of *TP53* mutation is highest in anaplastic carcinoma compared with other types of aggressive/advanced thyroid tumors, including poorly differentiated and high-grade differentiated carcinomas. Unlike *TERT* promoter mutation (see below), the impact of *TP53* loss-of-function on survival is not independent of histological type, and in combined tumors, it is associated with the anaplastic component (14, 19, 20, 37, 52, 124, 126, 150, 151). Mechanistically, the effect of p53 disruption in thyroid tumors was initially investigated using preclinical *in vitro* and *in vivo* models focusing on DNA repair, thyroid dedifferentiation and ATC transformation (Figs 1 and 3 and Table 3). Studies have demonstrated that DNA repair in irradiated thyroid cancer cells is impaired in *TP53*-mutant compared to *TP53*-wild type cells (152). *TP53*-mutated thyroid cell lines are also characterized by loss of the PAX8 thyroid differentiation transcription factor, which is restored upon reintroduction of wild type *TP53* (153). Numerous additional studies have shown the effect of *TP53* disruption in thyroid cancer progression when it is co-mutated with early oncogenic events, such as *RET* fusion (154), *BRAF* p.V600E (155, 156, 157), *HRAS* p.G12V (*158*), *KRAS* p.G12D (*159*)*, PTEN* mutation (143), and *STRN::ALK* fusion (160). Dedifferentiation, EMT, uncontrolled tumor growth, and metastases were the main reported outcomes (143, 154, 155, 156, 157, 158, 159, 160). Remarkably, upon p53 loss, *BRAF* p.V600E-driven thyroid carcinoma transgenic mice show overactivation of MAPK and PI3K signaling and immunosuppressive tumor-associated macrophages, two common features of anaplastic carcinoma (155, 156, 157). Taken together, both clinical and laboratory evidence establish the relevance of *TP53* disruption in progression to anaplastic carcinoma.

### *TERT* promoter mutation

*TERT* encodes telomerase reverse transcriptase, the enzymatic core protein of the telomerase complex that maintains telomere length at chromosomal ends by adding the TTAGGG repeat. The telomerase complex controls cellular senescence. It is normally repressed in postnatal adult somatic cells leading to progressive telomere shortening and cell death, but remains physiologically active only in the ‘immortal’ stem cell compartments of self-renewing tissues (161, 162, 163, 164). *TERT* promoter mutations enable TERT re-expression, otherwise repressed in adult normal cells, inhibiting neoplastic cell senescence and death. The mechanism through which this occurs was unraveled in 2013 when two seminal articles showed that a high proportion of melanomas have mutually exclusive mutations at two hotspots in the *TERT* promoter (positions −124, C228T and −146, C250T) that create novel binding sites for ETS (erythroblast transformation specific) family transcription factors, thereby promoting *TERT* transcription (165, 166). ETS transcription factors are involved with a wide variety of functions, and their expression is also induced by MAPK signaling (e.g., after activation by *RAS* or *BRAF*). As *TERT* promoter mutations may be associated with *BRAF* p.V600E or *RAS* mutations, co-mutation has the potential to further increase *TERT* transcription in *BRAF*- or *RAS*-mutated tumors (165).

Since 2013, C228T and C250T *TERT* promoter mutations have been found in many other tumor types originating from organs with a stable population of cells that do not undergo constant turnover and where telomerase is not normally expressed (167). In the thyroid gland, they were identified in tumors of follicular cells and in medullary carcinoma. Although rare in the latter (41), they have a high prevalence in follicular cell tumors with distant metastases, of advanced stage and poor outcome (Figs 1 and 3 and Table 3) (14, 19, 20, 37, 52, 168, 169, 170, 171). The highest prevalence occurs in anaplastic carcinoma, consistent with the important role of *TERT* promoter mutation as a marker of disease progression. However, unlike *TP53* mutation, the other established progression marker, *TERT* promoter mutations are associated with distant metastases independently of tumor histology. Indeed, they have been identified in metastatic tumors that are well differentiated and histologically low grade (37, 170, 172). In particular, the THYT1 signature, a combination of *TERT* promoter mutation, duplication of Chr5p (harboring the *TERT* genomic locus) and duplication of Chr1q (a chromosomal site specifically associated with poor prognosis in papillary carcinoma (11), any of these three alterations), has been proposed as a marker for metastatic spread and poor outcome in papillary carcinoma (15). Since MAPK activation in *BRAF* p.V600E- or *RAS*-mutated tumors increases *TERT* transcription, *BRAF* p.V600E or *RAS* co-mutation with *TERT* has been strongly associated with advanced disease and poor outcome, even in aggressive tumors, such as high grade non-anaplastic (12) and anaplastic carcinoma (38).

Overall, strong clinical evidence establishes *TERT* promoter mutation as a very useful marker for risk stratification to predict the outcome in thyroid carcinomas of follicular cells (14, 19, 20, 37, 52, 168, 169, 170, 171).

### *SWI/SNF* alteration

The SWI/SNF chromatin remodeling complex, in humans referred to as the BAF (BRG1/BR-associated factor, or mammalian SWI/SNF) complex, is an ATP-dependent multi-subunit protein system that modulates chromatin structure by modifying DNA–nucleosome interaction to enable chromatin accessibility for gene transcription (173). SWI/SNF proteins slide nucleosomes on DNA or evict nucleosomic histones. Hence, they are referred to as “access remodellers” that promote gene expression by exposing binding sites to transcription factors (173). Loss-of-function mutations in specific SWI/SNF proteins reprogram chromatin structure by restricting promoter access to transcription factors that enforce cell differentiation, promoting an undifferentiated stem-cell-like phenotype. They occur in approximately 20% of human neoplasms, in a tissue-specific manner. For example, *ARID1A* mutations are identified in subsets of endometrial, ovarian, bladder, gastric, liver and biliopancreatic carcinomas (173, 174, 175). Due to the tumorigenic role of SWI/SNF proteins, small molecules that inactivate the SWI/SNF complex or cause protein degradation are being evaluated in preclinical studies, and are under investigation in clinical trials designed for patients with SWI/SNF-mutated tumors (173). In thyroid carcinoma, alteration of the SWI/SNF complex is typically due to *ARID1A*, *ARID1B*, *ARID2*, *SMARCB1* or *PBRM1* mutations. These occur as secondary co-mutations in tumors carrying early/driver molecular changes. They are collectively identified in approximately 10% of high-grade non-anaplastic carcinomas (HGDTC and PDTC) and in up to 15–35% of anaplastic carcinomas, but rarely found in low grade papillary or follicular carcinomas, indicating their role in progression to aggressive and anaplastic forms of cancer (Figs 1 and 3 and Table 3) (12, 14, 38, 52).

The role of SWI/SNF disruption in thyroid cancer progression has been investigated in transgenic mice with co-mutation of SWI/SNF genes and *HRAS* p.G12V or *BRAF* p.V600E, highlighting the cooperative role of SWI/SNF in promoting loss of differentiation and tumor progression (176, 177). SWI\SNF loss-of-function locks *BRAF* p.V600E-driven thyroid mice tumors into a dedifferentiated state. In these models, MAPK inhibitor-based redifferentiation is ineffective in restoring differentiated thyroid function and radioiodine uptake (177). Indeed, the results of a pilot clinical trial using a MAPK inhibition redifferentiation strategy in patients with *BRAF* p.V600E radioiodine-refractory thyroid carcinoma suggests that SWI/SNF gene mutation may be a marker of resistance to these forms of treatment (178).

### Other alterations associated with aggressive thyroid carcinoma

Disruption of DNA repair occurs during tumor progression. Indeed, loss-of-function of DNA mismatch repair pathway genes (*MLH1*, *MSH2* and *MSH6*), and of the *ATM* (Ataxia-telangiectasia mutated) gene involved with both homologous recombination and non-homologous end-joining DNA repair, have been found – typically as co-mutations – in high grade, aggressive tumors. Mismatch repair genes mutation has been reported in up to approximately 10% of anaplastic carcinomas, but is rare in high grade non-anaplastic carcinomas (HGDTC and PDTC) (12, 14, 38, 52). *ATM* mutations have been reported in approximately 5% of high grade non-anaplastic tumors (HGDTC and PDTC) and in approximately 5–10% of anaplastic carcinomas (12, 14, 38, 52).

*NF2*, encoding for Merlin, a component of the Hippo pathway (179), is mutated, in up to approximately 10% of anaplastic carcinomas, but rarely in high grade non-anaplastic carcinomas (HGDTC and PDTC). Mutations are loss-of function, secondary events (38, 180).

Loss-of-function of RBM10 – a regulator of alternative splicing – occurs, typically as co-mutation, in up to approximately 5% of high grade non-anaplastic carcinomas (HGDTC and PDTC) and in a small subset of anaplastic tumors (12, 14, 38, 52, 181).

Loss-of-function secondary mutations of histone methyltransferases (KMT2A, KMT2C, KMT2D and SETD2) and histone acetyltransferases (CREBBP and EP300) have been reported in small subsets of high grade non-anaplastic carcinomas (HGDTC and PDTC) and of anaplastic tumors (12, 14, 38, 52).

## Conclusion

The genetic molecular pathology of thyroid tumors of follicular cells has come into focus in the recent past due to high throughput technology. Understanding the genetic landscape and related molecular mechanisms underlying tumor development and progression is an unavoidable step to improve the clinical management of thyroid nodules and to find suitable molecular targets for patients affected by aggressive and advanced forms of carcinoma. In brief, somatic genetic alterations of thyroid tumors of follicular cells are characterized by the following:

- Tumors of follicular cells feature a remarkable genotype–phenotype correlation.
- Primary and mutually exclusive early/driver molecular alterations affecting the MAPK pathway are involved in tumor development and characterize well-differentiated carcinomas, while secondary events are required for progression to aggressive high grade differentiated, poorly differentiated and undifferentiated forms of carcinoma.
- Primary molecular alterations are *RAS* or RAS-like for follicular-patterned tumors, *BRAF* p.V600E or BRAF V600E-like (including rearrangement of tyrosine kinase, such as *RET* and *NTRK)* for conventional papillary carcinomas, while other genetic alterations, specifically somatic mtDNA mutations and genome haploidization, characterize oncocytic tumors.
- Progression of thyroid tumors into aggressive and less-differentiated forms depends on the acquisition of secondary oncogenic co-mutations in addition to the primary BRAF V600E-like, RAS-like or other driver event. These additional alterations, other than MAPK hyperactivity, include *PTEN* or *PIK3CA* mutation driven aberrant PI3K–PTEN–AKT signaling, cell cycle dysregulation due to *TP53* loss-of-function, *TERT* promoter mutations inhibiting neoplastic cell senescence and death, and SWI–SNF dependent chromatin reconfiguration.

## Declaration of interest

The authors declare that there is no conflict of interest that could be perceived as prejudicing the impartiality of the work reported.

## Funding

The work reported in this publication was funded by the Italian Ministry of Health, RC-2024-2790136.

## Author contribution statement

Conceptualization was performed by GC, FJDP, and GT. GC, FJDP, and GT helped in writing of the original draft. Visualization was performed by GC and FJDP. Investigation and data curation was performed by GC, FJDP, ADL, TM, SCo, LP, AR, ES, SD, SCh, FC, DdB, and GT. Supervision was performed by GT, DdB, and ADL; funding acquisition was performed by GT. GC, FJDP, KJR, and GT helped in writing of the review and editing.

## References

[1] International Agency for Research on Cancer. Cancer today. Lyon, France: Global Cancer Observatory, 2024. (https://gco.iarc.fr/today/en2024)

[2] Boucai L, Zafereo M & Cabanillas ME. Thyroid cancer: a review. JAMA 2024 331 425–435. (10.1001/jama.2023.26348) 38319329

[3] Lyu Z, Zhang Y, Sheng C, et al. Global burden of thyroid cancer in 2022: incidence and mortality estimates from GLOBOCAN. Chin Med J Engl 2024 137 2567–2576. (10.1097/cm9.0000000000003284) 39261986 PMC11557048

[4] Wang C, Wu Z, Lei L, et al. Geographic disparities in trends of thyroid cancer incidence and mortality from 1990 to 2019 and a projection to 2030 across income-classified countries and territories. J Glob Health 2023 13 04108. (10.7189/jogh.13.04108) 37766638 PMC10540248

[5] Li M, Maso LD & Vaccarella S. Global trends in thyroid cancer incidence and the impact of overdiagnosis. Lancet Diabetes Endocrinol 2020 8 468–470. (10.1016/s2213-8587(20)30115-7) 32445733

[6] Baloch ZW, Asa SL, Barletta JA, et al. Overview of the 2022 WHO classification of thyroid neoplasms. Endocr Pathol 2022 33 27–63. (10.1007/s12022-022-09707-3) 35288841

[7] Haugen BR, Alexander EK, Bible KC, et al. 2015 American Thyroid Association Management Guidelines for adult patients with thyroid nodules and differentiated thyroid cancer: the American Thyroid Association Guidelines task force on thyroid nodules and differentiated thyroid cancer. Thyroid 2016 26 1–133. (10.1089/thy.2015.0020) 26462967 PMC4739132

[8] Bible KC, Kebebew E, Brierley J, et al. 2021 American Thyroid Association Guidelines for management of patients with anaplastic thyroid cancer. Thyroid 2021 31 337–386. (10.1089/thy.2020.0944) 33728999 PMC8349723

[9] Fagin JA & Wells SA. Biologic and clinical perspectives on thyroid cancer. N Engl J Med 2016 375 1054–1067. (10.1056/nejmra1501993) 27626519 PMC5512163

[10] Volante M, Collini P, Nikiforov YE, et al. Poorly differentiated thyroid carcinoma: the Turin proposal for the use of uniform diagnostic criteria and an algorithmic diagnostic approach. Am J Surg Pathol 2007 31 1256–1264. (10.1097/pas.0b013e3180309e6a) 17667551

[11] Wong KS, Dong F, Telatar M, et al. Papillary thyroid carcinoma with high-grade features versus poorly differentiated thyroid carcinoma: an analysis of clinicopathologic and molecular features and outcome. Thyroid 2021 31 933–940. (10.1089/thy.2020.0668) 33143568

[12] Xu B, David J, Dogan S, et al. Primary high-grade non-anaplastic thyroid carcinoma: a retrospective study of 364 cases. Histopathology 2022 80 322–337. (10.1111/his.14550) 34449926 PMC9425734

[13] Barletta JA, Nosé V & Sadow PM. Genomics and epigenomics of medullary thyroid carcinoma: from sporadic disease to familial manifestations. Endocr Pathol 2021 32 35–43. (10.1007/s12022-021-09664-3) 33492588 PMC9353617

[14] Landa I, Ibrahimpasic T, Boucai L, et al. Genomic and transcriptomic hallmarks of poorly differentiated and anaplastic thyroid cancers. J Clin Investig 2016 126 1052–1066. (10.1172/jci85271) 26878173 PMC4767360

[15] Gandolfi G, Ragazzi M, de Biase D, et al. Genome-wide profiling identifies the THYT1 signature as a distinctive feature of widely metastatic papillary thyroid carcinomas. Oncotarget 2017 9 1813–1825. (10.18632/oncotarget.22805) 29416733 PMC5788601

[16] Chen H, Luthra R, Routbort MJ, et al. Molecular profile of advanced thyroid carcinomas by next-generation sequencing: characterizing tumors beyond diagnosis for targeted therapy. Mol Cancer Ther 2018 17 1575–1584. (10.1158/1535-7163.mct-17-0871) 29695638

[17] Cabanillas ME, Ryder M & Jimenez C. Targeted therapy for advanced thyroid cancer: kinase inhibitors and beyond. Endocr Rev 2019 40 1573–1604. (10.1210/er.2019-00007) 31322645 PMC7341904

[18] Fagin JA, Krishnamoorthy GP & Landa I. Pathogenesis of cancers derived from thyroid follicular cells. Nat Rev Cancer 2023 23 631–650. (10.1038/s41568-023-00598-y) 37438605 PMC10763075

[19] Acquaviva G, Visani M, Repaci A, et al. Molecular pathology of thyroid tumours of follicular cells: a review of genetic alterations and their clinicopathological relevance. Histopathology 2018 72 6–31. (10.1111/his.13380) 29239040

[20] De Leo A, Ruscelli M, Maloberti T, et al. Molecular pathology of endocrine gland tumors: genetic alterations and clinicopathologic relevance. Virchows Arch 2024 484 289–319. (10.1007/s00428-023-03713-4) 38108848 PMC10948534

[21] Cancer Genome Atlas Research Network. Integrated genomic characterization of papillary thyroid carcinoma. Cell 2014 159 676–690. (10.1016/j.cell.2014.09.050) 25417114 PMC4243044

[22] Mitsutake N, Knauf JA, Mitsutake S, et al. Conditional BRAFV600E expression induces DNA synthesis, apoptosis, dedifferentiation, and chromosomal instability in thyroid PCCL3 cells. Cancer Res 2005 65 2465–2473. (10.1158/0008-5472.can-04-3314) 15781663

[23] Veschi V, Turdo A, Modica C, et al. Recapitulating thyroid cancer histotypes through engineering embryonic stem cells. Nat Commun 2023 14 1351. (10.1038/s41467-023-36922-1) 36906579 PMC10008571

[24] Bonora E, Porcelli AM, Gasparre G, et al. Defective oxidative phosphorylation in thyroid oncocytic carcinoma is associated with pathogenic mitochondrial DNA mutations affecting complexes I and III. Cancer Res 2006 66 6087–6096. (10.1158/0008-5472.can-06-0171) 16778181

[25] Gasparre G, Porcelli AM, Bonora E, et al. Disruptive mitochondrial DNA mutations in complex I subunits are markers of oncocytic phenotype in thyroid tumors. Proc Natl Acad Sci U S A 2007 104 9001–9006. (10.1073/pnas.0703056104) 17517629 PMC1885617

[26] Corver WE, Ruano D, Weijers K, et al. Genome haploidisation with chromosome 7 retention in oncocytic follicular thyroid carcinoma. PLoS One 2012 7 e38287. (10.1371/journal.pone.0038287) 22675538 PMC3365880

[27] Corver WE, van Wezel T, Molenaar K, et al. Near-haploidization significantly associates with oncocytic adrenocortical, thyroid, and parathyroid tumors but not with mitochondrial DNA mutations. Genes Chromosomes Cancer 2014 53 833–844. (10.1002/gcc.22194) 24909752

[28] Ganly I, Makarov V, Deraje S, et al. Integrated genomic analysis of hürthle cell cancer reveals oncogenic drivers, recurrent mitochondrial mutations, and unique chromosomal landscapes. Cancer Cell 2018 34 256–270.e5. (10.1016/j.ccell.2018.07.002) 30107176 PMC6247912

[29] Gopal RK, Kübler K, Calvo SE, et al. Widespread chromosomal losses and mitochondrial DNA alterations as genetic drivers in Hürthle cell carcinoma. Cancer Cell 2018 34 242–255.e5. (10.1016/j.ccell.2018.06.013) 30107175 PMC6121811

[30] Quinlan M & Settleman J. Isoform-specific ras functions in development and cancer. Future Oncol 2009 5 105–116. (10.2217/14796694.5.1.105) 19243303

[31] Castellano E & Santos E. Functional specificity of ras isoforms: so similar but so different. Genes Cancer 2011 2 216–231. (10.1177/1947601911408081) 21779495 PMC3128637

[32] Prior IA, Lewis PD & Mattos C. A comprehensive survey of ras mutations in cancer. Cancer Res 2012 72 2457–2467. (10.1158/0008-5472.can-11-2612) 22589270 PMC3354961

[33] Nikiforova MN, Lynch RA, Biddinger PW, et al. RAS point mutations and PAX8-PPAR gamma rearrangement in thyroid tumors: evidence for distinct molecular pathways in thyroid follicular carcinoma. J Clin Endocrinol Metab 2003 88 2318–2326. (10.1210/jc.2002-021907) 12727991

[34] Vasko V, Ferrand M, Di Cristofaro J, et al. Specific pattern of RAS oncogene mutations in follicular thyroid tumors. J Clin Endocrinol Metab 2003 88 2745–2752. (10.1210/jc.2002-021186) 12788883

[35] Nikiforov YE, Seethala RR, Tallini G, et al. Nomenclature revision for encapsulated follicular variant of papillary thyroid carcinoma a paradigm shift to reduce overtreatment of indolent tumors. JAMA Oncol 2016 2 1023–1029. (10.1001/jamaoncol.2016.0386) 27078145 PMC5539411

[36] Seethala RR, Baloch ZW, Barletta JA, et al. Noninvasive follicular thyroid neoplasm with papillary-like nuclear features: a review for pathologists. Mod Pathol 2018 31 39–55. (10.1038/modpathol.2017.130) 29052599

[37] Volante M, Lam AK, Papotti M, et al. Molecular pathology of poorly differentiated and anaplastic thyroid cancer: what do pathologists need to know? Endocr Pathol 2021 32 63–76. (10.1007/s12022-021-09665-2) 33543394 PMC7960587

[38] Xu B, Fuchs T, Dogan S, et al. Dissecting anaplastic thyroid carcinoma: a comprehensive clinical, histologic, immunophenotypic, and molecular study of 360 cases. Thyroid 2020 30 1505–1517. (10.1089/thy.2020.0086) 32284020 PMC7583343

[39] Boichard A, Croux L, Al Ghuzlan A, et al. Somatic RAS mutations occur in a large proportion of sporadic RET-negative medullary thyroid carcinomas and extend to a previously unidentified exon. J Clin Endocrinol Metab 2012 97 E2031–E2035. (10.1210/jc.2012-2092) 22865907 PMC3462939

[40] Ciampi R, Mian C, Fugazzola L, et al. Evidence of a low prevalence of RAS mutations in a large medullary thyroid cancer series. Thyroid 2013 23 50–57. (10.1089/thy.2012.0207) 23240926

[41] Xu B, Viswanathan K, Ahadi MS, et al. Association of the genomic profile of medullary thyroid carcinoma with tumor characteristics and clinical outcomes in an international multicenter study. Thyroid 2024 34 167–176. (10.1089/thy.2023.0279) 37842841 PMC10884546

[42] Cohen DS, Tongson-Ignacio JE, Lolachi CM, et al. Rethinking malignancy risk in indeterminate thyroid nodules with positive molecular studies: southern California permanente experience. Otolaryngol Head Neck Surg 2019 161 419–423. (10.1177/0194599819842859) 31013183

[43] Filetti S, Durante C, Hartl D, et al. Thyroid cancer: ESMO clinical practice guidelines for diagnosis, treatment and follow-up. Ann Oncol 2019 30 1856–1883. (10.1093/annonc/mdz400) 31549998

[44] Kroll TG, Sarraf P, Pecciarini L, et al. *PAX8-PPAR* γ *1* fusion in oncogene human thyroid carcinoma. Science 2000 289 1357–1360. (10.1126/science.289.5483.1357) 10958784

[45] Raman P & Koenig RJ. Pax-8-PPAR-γ fusion protein in thyroid carcinoma. Nat Rev Endocrinol 2014 10 616–623. (10.1038/nrendo.2014.115) 25069464 PMC4290886

[46] Nicolson NG, Murtha TD, Dong W, et al. Comprehensive genetic analysis of follicular thyroid carcinoma predicts prognosis independent of histology. J Clin Endocrinol Metab 2018 103 2640–2650. (10.1210/jc.2018-00277) 29726952

[47] Lui WO, Zeng L, Rehrmann V, et al. CREB3L2-PPARγ fusion mutation identifies a thyroid signaling pathway regulated by intramembrane proteolysis. Cancer Res 2008 68 7156–7164. (10.1158/0008-5472.can-08-1085) 18757431

[48] Dobson ME, Diallo-Krou E, Grachtchouk V, et al. Pioglitazone induces a proadipogenic antitumor response in mice with PAX8-PPARγ fusion protein thyroid carcinoma. Endocrinology 2011 152 4455–4465. (10.1210/en.2011-1178) 21952241 PMC3199014

[49] Karunamurthy A, Panebianco F, Hsiao SJ, et al. Prevalence and phenotypic correlations of EIF1AX mutations in thyroid nodules. Endocr Relat Cancer 2016 23 295–301. (10.1530/erc-16-0043) 26911375 PMC5494715

[50] Krishnamoorthy GP, Davidson NR, Leach SD, et al. EIF1AX and RAS mutations cooperate to drive thyroid tumorigenesis through ATF4 and c-MYC. Cancer Discov 2019 9 264–281. (10.1158/2159-8290.cd-18-0606) 30305285 PMC6373451

[51] Abi-Raad R, Xu B, Gilani S, et al. EIF1AX mutation in thyroid nodules: a histopathologic analysis of 56 cases in the context of institutional practices. Virchows Arch 2024 485 859–867. (10.1007/s00428-024-03914-5) 39225726 PMC11912518

[52] Pozdeyev N, Gay LM, Sokol ES, et al. Genetic analysis of 779 advanced differentiated and anaplastic thyroid cancers. Clin Cancer Res 2018 24 3059–3068. (10.1158/1078-0432.ccr-18-0373) 29615459 PMC6030480

[53] Davies H, Bignell GR, Cox C, et al. Mutations of the BRAF gene in human cancer. Nature 2002 417 949–954. (10.1038/nature00766) 12068308

[54] Nikiforov YE & Nikiforova MN. Molecular genetics and diagnosis of thyroid cancer. Nat Rev Endocrinol 2011 7 569–580. (10.1038/nrendo.2011.142) 21878896

[55] Durante C, Puxeddu E, Ferretti E, et al. BRAF mutations in papillary thyroid carcinomas inhibit genes involved in iodine metabolism. J Clin Endocrinol Metab 2007 92 2840–2843. (10.1210/jc.2006-2707) 17488796

[56] Tallini G, Tuttle RM & Ghossein RA. The history of the follicular variant of papillary thyroid carcinoma. J Clin Endocrinol Metab 2017 102 15–22. (10.1210/jc.2016-2976) 27732333

[57] Xing M, Westra WH, Tufano RP, et al. BRAF mutation predicts a poorer clinical prognosis for papillary thyroid cancer. J Clin Endocrinol Metab 2005 90 6373–6379. (10.1210/jc.2005-0987) 16174717

[58] Elisei R, Viola D, Torregrossa L, et al. The BRAF(V600E) mutation is an independent, poor prognostic factor for the outcome of patients with low-risk intrathyroid papillary thyroid carcinoma: single-institution results from a large cohort study. J Clin Endocrinol Metab 2012 97 4390–4398. (10.1210/jc.2012-1775) 23066120

[59] Trovisco V, Soares P, Preto A, et al. Type and prevalence of BRAF mutations are closely associated with papillary thyroid carcinoma histotype and patients’ age but not with tumour aggressiveness. Virchows Arch 2005 446 589–595. (10.1007/s00428-005-1236-0) 15902486

[60] Yasuhiro I, Yoshida H, Maruo R, et al. BRAF mutation in papillary thyroid carcinoma in a Japanese population: its lack of correlation with high-risk clinicopathological features and disease-free survival of patients. Endocr J 2009 56 89–97. (10.1507/endocrj.k08e-208) 18840924

[61] Sancisi V, Nicoli D, Ragazzi M, et al. BRAFV600E mutation does not mean distant metastasis in thyroid papillary carcinomas. J Clin Endocrinol Metab 2012 97 E1745–E1749. (10.1210/jc.2012-1526) 22740704

[62] Romei C, Ciampi R, Faviana P, et al. BRAFV600E mutation, but not RET/PTC rearrangements, is correlated with a lower expression of both thyroperoxidase and sodium iodide symporter genes in papillary thyroid cancer. Endocr Relat Cancer 2008 15 511–520. (10.1677/erc-07-0130) 18509003

[63] Zheng X, Wei S, Han Y, et al. Papillary microcarcinoma of the thyroid: clinical characteristics and BRAF(V600E) mutational status of 977 cases. Ann Surg Oncol 2013 20 2266–2273. (10.1245/s10434-012-2851-z) 23370668

[64] Walczyk A, Kowalska A, Kowalik A, et al. The BRAF(V600E) mutation in papillary thyroid microcarcinoma: does the mutation have an impact on clinical outcome? Clin Endocrinol 2014 80 899–904. (10.1111/cen.12386) 24354346

[65] Xing M, Alzahrani AS, Carson KA, et al. Association between BRAF V600E mutation and recurrence of papillary thyroid cancer. J Clin Oncol 2015 33 42–50. (10.1200/jco.2014.56.8253) 25332244 PMC4268252

[66] Tavares C, Melo M, Cameselle-Teijeiro JM, et al. Endocrine tumours: genetic predictors of thyroid cancer outcome. Eur J Endocrinol 2016 174 R117–R126. (10.1530/eje-15-0605) 26510840

[67] Torregrossa L, Viola D, Sensi E, et al. Papillary thyroid carcinoma with rare Exon 15 BRAF mutation has indolent behavior: a single-institution experience. J Clin Endocrinol Metab 2016 101 4413–4420. (10.1210/jc.2016-1775) 27571181

[68] Acquaviva G, de Biase D, Diquigiovanni C, et al. BRAF exon 15 mutations in papillary carcinoma and adjacent thyroid parenchyma: a search for the early molecular events associated with tumor development. Cancers 2020 12 430. (10.3390/cancers12020430) 32059434 PMC7072486

[69] De Leo A, Serban D, Maloberti T, et al. Expanding the spectrum of BRAF Non-V600E mutations in thyroid nodules: evidence-based data from a tertiary referral centre. Int J Mol Sci 2023 24 4057. (10.3390/ijms24044057) 36835466 PMC9958561

[70] Yao Z, Yaeger R, Rodrik-Outmezguine VS, et al. Tumours with class 3 BRAF mutants are sensitive to the inhibition of activated RAS. Nature 2017 548 234–238. (10.1038/nature23291) 28783719 PMC5648058

[71] Santoro M, Moccia M, Federico G, et al. RET gene fusions in malignancies of the thyroid and other tissues. Genes 2020 11 424. (10.3390/genes11040424) 32326537 PMC7230609

[72] Prasad ML, Vyas M, Horne MJ, et al. NTRK fusion oncogenes in pediatric papillary thyroid carcinoma in northeast United States. Cancer 2016 122 1097–1107. (10.1002/cncr.29887) 26784937

[73] Chu YH, Wirth LJ, Farahani AA, et al. Clinicopathologic features of kinase fusion-related thyroid carcinomas: an integrative analysis with molecular characterization. Mod Pathol 2020 33 2458–2472. (10.1038/s41379-020-0638-5) 32737449 PMC7688509

[74] Pekova B, Sykorova V, Dvorakova S, et al. RET, NTRK, ALK, BRAF, and MET fusions in a large cohort of pediatric papillary thyroid carcinomas. Thyroid 2020 30 1771–1780. (10.1089/thy.2019.0802) 32495721

[75] Chu YH & Sadow PM. Kinase fusion-related thyroid carcinomas: towards predictive models for advanced actionable diagnostics. Endocr Pathol 2022 33 421–435. (10.1007/s12022-022-09739-9) 36308634 PMC10283356

[76] Chou A, Qiu MR, Crayton H, et al. A detailed histologic and molecular assessment of the diffuse sclerosing variant of papillary thyroid carcinoma. Mod Pathol 2023 36 100329. (10.1016/j.modpat.2023.100329) 37716505

[77] Scholfield DW, Fitzgerald CWR, Boe LA, et al. Defining the genomic landscape of diffuse sclerosing papillary thyroid carcinoma: prognostic implications of RET fusions. Ann Surg Oncol 2024 31 5525–5536. (10.1245/s10434-024-15500-9) 38847983 PMC12416437

[78] Bongarzone I, Vigneri P, Mariani L, et al. RET/NTRK1 rearrangements in thyroid gland tumors of the papillary carcinoma family: correlation with clinicopathological features. Clin Cancer Res 1998 4 223–228. 9516975

[79] Bongarzone I, Pierotti MA, Monzini N, et al. High frequency of activation of tyrosine kinase oncogenes in human papillary thyroid carcinoma. Oncogene 1989 4 1457–1462. 2594368

[80] Kelly LM, Barila G, Liu P, et al. Identification of the transforming STRN-ALK fusion as a potential therapeutic target in the aggressive forms of thyroid cancer. Proc Natl Acad Sci U S A 2014 111 4233–4238. (10.1073/pnas.1321937111) 24613930 PMC3964116

[81] Russell JP, Powell DJ, Cunnane M, et al. The TRK-T1 fusion protein induces neoplastic transformation of thyroid epithelium. Oncogene 2000 19 5729–5735. (10.1038/sj.onc.1203922) 11126359

[82] Nikitski AV, Rominski SL, Wankhede M, et al. Mouse model of poorly differentiated thyroid carcinoma driven by STRN-ALK fusion. Am J Pathol 2018 188 2653–2661. (10.1016/j.ajpath.2018.07.012) 30125543 PMC6222272

[83] Pérot G, Soubeyran I, Ribeiro A, et al. Identification of a recurrent STRN/ALK fusion in thyroid carcinomas. PLoS One 2014 9 e87170. (10.1371/journal.pone.0087170) 24475247 PMC3903624

[84] Hamatani K, Mukai M, Takahashi K, et al. Rearranged anaplastic lymphoma kinase (ALK) gene in adult-onset papillary thyroid cancer amongst atomic bomb survivors. Thyroid 2012 22 1153–1159. (10.1089/thy.2011.0511) 23050789 PMC3487115

[85] Chong AS, Nikiforov YE, Condello V, et al. Prevalence and spectrum of DICER1 mutations in adult-onset thyroid nodules with indeterminate cytology. J Clin Endocrinol Metab 2021 106 968–977. (10.1210/clinem/dgab025) 33460435

[86] Foulkes WD, Priest JR & Duchaine TF. DICER1: mutations, microRNAs and mechanisms. Nat Rev Cancer 2014 14 662–672. (10.1038/nrc3802) 25176334

[87] Ricarte-Filho JC, Casado-Medrano V, Reichenberger E, et al. DICER1 RNase IIIb domain mutations trigger widespread miRNA dysregulation and MAPK activation in pediatric thyroid cancer. Front Endocrinol 2023 14 1083382. (10.3389/fendo.2023.1083382) PMC9990750 36896180

[88] Minna E, Devecchi A, Pistore F, et al. Genomic and transcriptomic analyses of thyroid cancers identify DICER1 somatic mutations in adult follicular-patterned RAS-like tumors. Front Endocrinol 2023 14 1267499. (10.3389/fendo.2023.1267499) PMC10585144 37867524

[89] Costa V, Esposito R, Ziviello C, et al. New somatic mutations and WNK1-B4GALNT3 gene fusion in papillary thyroid carcinoma. Oncotarget 2015 6 11242–11251. (10.18632/oncotarget.3593) 25803323 PMC4484453

[90] Yoo SK, Lee S, Kim SJ, et al. Comprehensive analysis of the transcriptional and mutational landscape of follicular and papillary thyroid cancers. PLoS Genet 2016 12 e1006239. (10.1371/journal.pgen.1006239) 27494611 PMC4975456

[91] Wasserman JD, Sabbaghian N, Fahiminiya S, et al. DICER1 mutations are frequent in adolescent-onset papillary thyroid carcinoma. J Clin Endocrinol Metab 2018 103 2009–2015. (10.1210/jc.2017-02698) 29474644

[92] Bongiovanni M, Sykiotis GP, La Rosa S, et al. Macrofollicular variant of follicular thyroid carcinoma: a rare underappreciated pitfall in the diagnosis of thyroid carcinoma. Thyroid 2020 30 72–80. (10.1089/thy.2018.0607) 31701808

[93] Chernock RD, Rivera B, Borrelli N, et al. Poorly differentiated thyroid carcinoma of childhood and adolescence: a distinct entity characterized by DICER1 mutations. Mod Pathol 2020 33 1264–1274. (10.1038/s41379-020-0458-7) 31937902 PMC7329587

[94] Juhlin CC, Stenman A & Zedenius J. Macrofollicular variant follicular thyroid tumors are DICER1 mutated and exhibit distinct histological features. Histopathology 2021 79 661–666. (10.1111/his.14416) 34008223

[95] Ghossein CA, Dogan S, Farhat N, et al. Expanding the spectrum of thyroid carcinoma with somatic DICER1 mutation: a survey of 829 thyroid carcinomas using MSK-IMPACT next-generation sequencing platform. Virchows Arch 2022 480 293–302. (10.1007/s00428-021-03212-4) 34580763 PMC10126990

[96] Gallant JN, Chen SC, Ortega CA, et al. Evaluation of the molecular landscape of pediatric thyroid nodules and use of a multigene genomic classifier in children. JAMA Oncol 2022 8 1323–1327. (10.1001/jamaoncol.2022.1655) 35679040 PMC9185516

[97] Condello V, Poma AM, Macerola E, et al. Prevalence, molecular landscape, and clinical impact of DICER1 and DGCR8 mutated follicular-patterned thyroid nodules. J Clin Endocrinol Metab 2024 109 1733–1744. (10.1210/clinem/dgae034) 38252873 PMC11180504

[98] Condello V, Roberts JW, Stenman A, et al. Atrophic changes in thyroid tumors are strong indicators of underlying DICER1 mutations: a bi-institutional genotype-phenotype correlation study. Virchows Arch 2024 485 105–114. (10.1007/s00428-024-03802-y) 38637342 PMC11271315

[99] Juhlin CC & Mete O. Letter to the editor: morphological indicators of DICER1 mutations may guide somatic and germline testing. Thyroid 2025 35 120–121. (10.1089/thy.2024.0556) 39714925

[100] Agaimy A, Witkowski L, Stoehr R, et al. Malignant teratoid tumor of the thyroid gland: an aggressive primitive multiphenotypic malignancy showing organotypical elements and frequent DICER1 alterations-is the term “thyroblastoma” more appropriate? Virchows Arch 2020 477 787–798. (10.1007/s00428-020-02853-1) 32507920 PMC7683491

[101] Rooper LM, Bynum JP, Miller KP, et al. Recurrent DICER1 hotspot mutations in malignant thyroid gland teratomas: molecular characterization and proposal for a separate classification. Am J Surg Pathol 2020 44 826–833. (10.1097/pas.0000000000001430) 31917706

[102] Kock L, Wu MK & Foulkes WD. Ten years of DICER1 mutations: provenance, distribution, and associated phenotypes. Hum Mutat 2019 40 1939–1953. (10.1002/humu.23877) 31342592

[103] Juhlin CC. On the chopping block: overview of DICER1 mutations in endocrine and neuroendocrine neoplasms. Surg Pathol Clin 2023 16 107–118. (10.1016/j.path.2022.09.010) 36739158

[104] Altaraihi M, Hansen Tv O, Santoni-Rugiu E, et al. Prevalence of pathogenic germline DICER1 variants in young individuals thyroidectomised due to goitre – a national Danish cohort. Front Endocrinol 2021 12 727970. (10.3389/fendo.2021.727970) PMC8451242 34552563

[105] Tallini G, Ladanyi M, Rosai J, et al. Analysis of nuclear and mitochondrial DNA alterations in thyroid and renal oncocytic tumors. Cytogenet Cell Genet 1994 66 253–259. (10.1159/000133706) 7909283

[106] Tallini G, Hsueh A, Liu S, et al. Frequent chromosomal DNA unbalance in thyroid oncocytic (hürthle cell) neoplasms detected by comparative genomic hybridization. Lab Invest 1999 79 547–555. 10334566

[107] Máximo V, Soares P, Lima J, et al. Mitochondrial DNA somatic mutations (point mutations and large deletions) and mitochondrial DNA variants in human thyroid pathology: a study with emphasis on Hürthle cell tumors. Am J Pathol 2002 160 1857–1865. (10.1016/s0002-9440(10)61132-7) 12000737 PMC1850872

[108] Abi‐Raad R, Prasad ML, Adeniran AJ, et al. Copy number variations identified in thyroid FNA specimens are associated with Hürthle cell cytomorphology. Cancer Cytopathol 2022 130 415–422. (10.1002/cncy.22569) 35332982

[109] de Koster EJ, Corver WE, de Geus-Oei LF, et al. A clinically applicable molecular classification of oncocytic cell thyroid nodules. Endocr Relat Cancer 2023 30 e230047. (10.1530/erc-23-0047) 37399519 PMC10448578

[110] Flint A, Davenport RD, Lloyd RV, et al. Cytophotometric measurements of hürthle cell tumors of the thyroid gland. Correlation with pathologic features and clinical behavior. Cancer 1988 61 110–113. (10.1002/1097-0142(19880101)61:1<110::aid-cncr2820610119>3.0.co;2-4) 3334936

[111] Parma J, Duprez L, Sande JV, et al. Somatic mutations in the thyrotropin receptor gene cause hyperfunctioning thyroid adenomas. Nature 1993 365 649–651. (10.1038/365649a0) 8413627

[112] Trülzsch B, Krohn K, Wonerow P, et al. Detection of thyroid-stimulating hormone receptor and Gs α mutations: in 75 toxic thyroid nodules by denaturing gradient gel electrophoresis. J Mol Med Berl 2001 78 684–691. (10.1007/s001090000170) 11434721

[113] Gozu HI, Bircan R, Krohn K, et al. Similar prevalence of somatic TSH receptor and Gsα mutations in toxic thyroid nodules in geographical regions with different iodine supply in Turkey. Eur J Endocrinol 2006 155 535–545. (10.1530/eje.1.02253) 16990652

[114] Porcellini A, Fenzi G & Avvedimento EV. Mutations of thyrotropin receptor gene. J Mol Med Berl 1997 75 567–575. (10.1007/s001090050141) 9297624

[115] Calebiro D, Grassi ES, Eszlinger M, et al. Recurrent EZH1 mutations are a second hit in autonomous thyroid adenomas. J Clin Investig 2016 126 3383–3388. (10.1172/jci84894) 27500488 PMC5004945

[116] Cameselle-Teijeiro J & Chan JKC. Cribriform-morular variant of papillary carcinoma: a distinctive variant representing the sporadic counterpart of familial adenomatous polyposis-associated thyroid carcinoma? Mod Pathol 1999 12 400–411. 10229505

[117] Harach HR, Williams GT & Williams ED. Familial adenomatous polyposis associated thyroid carcinoma: a distinct type of follicular cell neoplasm. Histopathology 1994 25 549–561. (10.1111/j.1365-2559.1994.tb01374.x) 7698732

[118] Tomoda C, Miyauchi A, Uruno T, et al. Cribriform-morular variant of papillary thyroid carcinoma: clue to early detection of familial adenomatous polyposis-associated colon cancer. World J Surg 2004 28 886–889. (10.1007/s00268-004-7475-4) 15593462

[119] Cameselle-Teijeiro JM, Peteiro-González D, Caneiro-Gómez J, et al. Cribriform-morular variant of thyroid carcinoma: a neoplasm with distinctive phenotype associated with the activation of the WNT/β-catenin pathway. Mod Pathol 2018 31 1168–1179. (10.1038/s41379-018-0070-2) 29785019

[120] Boyraz B, Sadow PM, Asa SL, et al. Cribriform-morular thyroid carcinoma is a distinct thyroid malignancy of uncertain cytogenesis. Endocr Pathol 2021 32 327–335. (10.1007/s12022-021-09683-0) 34019236 PMC9353615

[121] Miyaki M, Iijima T, Ishii R, et al. Molecular evidence for multicentric development of thyroid carcinomas in patients with familial adenomatous polyposis. Am J Pathol 2000 157 1825–1827. (10.1016/s0002-9440(10)64821-3) 11106555 PMC1885783

[122] Xu B, Yoshimoto K, Miyauchi A, et al. Cribriform-morular variant of papillary thyroid carcinoma: a pathological and molecular genetic study with evidence of frequent somatic mutations in exon 3 of the beta-catenin gene. J Pathol 2003 199 58–67. (10.1002/path.1225) 12474227

[123] Shonka DC, Ho A, Chintakuntlawar AV, et al. American Head and Neck Society Endocrine Surgery Section and International Thyroid Oncology Group consensus statement on mutational testing in thyroid cancer: defining advanced thyroid cancer and its targeted treatment. Head Neck 2022 44 1277–1300. (10.1002/hed.27025) 35274388 PMC9332138

[124] Kunstman JW, Juhlin CC, Goh G, et al. Characterization of the mutational landscape of anaplastic thyroid cancer via whole-exome sequencing. Hum Mol Genet 2015 24 2318–2329. (10.1093/hmg/ddu749) 25576899 PMC4380073

[125] Dong W, Nicolson NG, Choi J, et al. Clonal evolution analysis of paired anaplastic and well-differentiated thyroid carcinomas reveals shared common ancestor. Genes Chromosomes Cancer 2018 57 645–652. (10.1002/gcc.22678) 30136351

[126] Ragazzi M, Torricelli F, Donati B, et al. Coexisting well-differentiated and anaplastic thyroid carcinoma in the same primary resection specimen: immunophenotypic and genetic comparison of the two components in a consecutive series of 13 cases and a review of the literature. Virchows Arch 2021 478 265–281. (10.1007/s00428-020-02891-9) 32683537

[127] Yoo SK, Song YS, Lee EK, et al. Integrative analysis of genomic and transcriptomic characteristics associated with progression of aggressive thyroid cancer. Nat Commun 2019 10 2764. (10.1038/s41467-019-10680-5) 31235699 PMC6591357

[128] Nguyen B, Fong C, Luthra A, et al. Genomic characterization of metastatic patterns from prospective clinical sequencing of 25,000 patients. Cell 2022 185 563–575.e11. (10.1016/j.cell.2022.01.003) 35120664 PMC9147702

[129] Pu W, Shi X, Yu P, et al. Single-cell transcriptomic analysis of the tumor ecosystems underlying initiation and progression of papillary thyroid carcinoma. Nat Commun 2021 12 6058. (10.1038/s41467-021-26343-3) 34663816 PMC8523550

[130] Lu L, Wang JR, Henderson YC, et al. Anaplastic transformation in thyroid cancer revealed by single-cell transcriptomics. J Clin Investig 2023 133 e169653. (10.1172/jci169653) 37053016 PMC10231997

[131] Hoxhaj G & Manning BD. The PI3K-AKT network at the interface of oncogenic signalling and cancer metabolism. Nat Rev Cancer 2020 20 74–88. (10.1038/s41568-019-0216-7) 31686003 PMC7314312

[132] García-Rostán G, Costa AM, Pereira-Castro I, et al. Mutation of the PIK3CA gene in anaplastic thyroid cancer. Cancer Res 2005 65 10199–10207. (10.1158/0008-5472.can-04-4259) 16288007

[133] Liu Z, Hou P, Ji M, et al. Highly prevalent genetic alterations in receptor tyrosine kinases and phosphatidylinositol 3-kinase/akt and mitogen-activated protein kinase pathways in anaplastic and follicular thyroid cancers. J Clin Endocrinol Metab 2008 93 3106–3116. (10.1210/jc.2008-0273) 18492751

[134] Ricarte-Filho JC, Ryder M, Chitale DA, et al. Mutational profile of advanced primary and metastatic radioactive iodine-refractory thyroid cancers reveals distinct pathogenetic roles for BRAF, PIK3CA, and AKT1. Cancer Res 2009 69 4885–4893. (10.1158/0008-5472.can-09-0727) 19487299 PMC2690720

[135] Ramírez-Moya J, Wert-Lamas L & Santisteban P. MicroRNA-146b promotes PI3K/AKT pathway hyperactivation and thyroid cancer progression by targeting PTEN. Oncogene 2018 37 3369–3383. (10.1038/s41388-017-0088-9) 29353884

[136] Miller KA, Yeager N, Baker K, et al. Oncogenic Kras requires simultaneous PI3K signaling to induce ERK activation and transform thyroid epithelial cells in vivo. Cancer Res 2009 69 3689–3694. (10.1158/0008-5472.can-09-0024) 19351816 PMC2669852

[137] Sponziello M, Lavarone E, Pegolo E, et al. Molecular differences between human thyroid follicular adenoma and carcinoma revealed by analysis of a murine model of thyroid cancer. Endocrinology 2013 154 3043–3053. (10.1210/en.2013-1028) 23751876 PMC3749486

[138] Jolly LA, Massoll N & Franco AT. Immune suppression mediated by Myeloid and lymphoid derived immune cells in the tumor microenvironment facilitates progression of thyroid cancers driven by HrasG12V and pten loss. J Clin Cell Immunol 2016 7 451. (10.4172/2155-9899.1000451) 27942419 PMC5145275

[139] Charles RP, Silva J, Iezza G, et al. Activating BRAF and PIK3CA mutations cooperate to promote anaplastic thyroid carcinogenesis. Mol Cancer Res 2014 12 979–986. (10.1158/1541-7786.mcr-14-0158-t) 24770869 PMC4635659

[140] Jolly LA, Novitskiy S, Owens P, et al. Fibroblast-mediated collagen remodeling within the tumor microenvironment facilitates progression of thyroid cancers driven by BrafV600E and pten loss. Cancer Res 2016 76 1804–1813. (10.1158/0008-5472.can-15-2351) 26818109 PMC4873339

[141] Shimamura M, Shibusawa N, Kurashige T, et al. Mouse models of sporadic thyroid cancer derived from BRAFV600E alone or in combination with PTEN haploinsufficiency under physiologic TSH levels. PLoS One 2018 13 e0201365. (10.1371/journal.pone.0201365) 30086162 PMC6080762

[142] Branigan GP, Casado-Medrano V, O’Neill AB, et al. Development of Novel Murine BRAFV600E-Driven papillary thyroid cancer cell lines for modeling of disease progression and preclinical evaluation of therapeutics. Cancers 2023 15 879. (10.3390/cancers15030879) 36765847 PMC9913801

[143] Arciuch VGA, Russo MA, Dima M, et al. Thyrocyte-specific inactivation of p53 and Pten results in anaplastic thyroid carcinomas faithfully recapitulating human tumors. Oncotarget 2011 2 1109–1126. (10.18632/oncotarget.380) 22190384 PMC3282070

[144] Jin N, Jiang T, Rosen DM, et al. Synergistic action of a RAF inhibitor and a dual PI3K/mTOR inhibitor in thyroid cancer. Clin Cancer Res 2011 17 6482–6489. (10.1158/1078-0432.ccr-11-0933) 21831957 PMC4828042

[145] ElMokh O, Ruffieux-Daidié D, Roelli MA, et al. Combined MEK and Pi3’-kinase inhibition reveals synergy in targeting thyroid cancer in vitro and in vivo. Oncotarget 2017 8 24604–24620. (10.18632/oncotarget.15599) 28445948 PMC5421873

[146] Bedard PL, Tabernero J, Janku F, et al. A phase Ib dose-escalation study of the oral pan-PI3K inhibitor buparlisib (BKM120) in combination with the oral MEK1/2 inhibitor trametinib (GSK1120212) in patients with selected advanced solid tumors. Clin Cancer Res 2015 21 730–738. (10.1158/1078-0432.ccr-14-1814) 25500057

[147] Sherr CJ & McCormick F. The RB and p53 pathways in cancer. Cancer Cell 2002 2 103–112. (10.1016/s1535-6108(02)00102-2) 12204530

[148] Kandoth C, McLellan MD, Vandin F, et al. Mutational landscape and significance across 12 major cancer types. Nature 2013 502 333–339. (10.1038/nature12634) 24132290 PMC3927368

[149] Mantovani F, Collavin L & Del Sal G. Mutant p53 as a guardian of the cancer cell. Cell Death Differ 2019 26 199–212. (10.1038/s41418-018-0246-9) 30538286 PMC6329812

[150] Dohi K, Seyama T, Mizuno T, et al. Unique association of p53 mutations with undifferentiated but not with differentiated carcinomas of the thyroid gland. Cancer Res 1992 52 1369–1371. 1737400

[151] Fagin JA, Matsuo K, Karmakar A, et al. High prevalence of mutations of the p53 gene in poorly differentiated human thyroid carcinomas. J Clin Investig 1993 91 179–184. (10.1172/jci116168) 8423216 PMC330012

[152] Yang TT, Namba H, Hara T, et al. p53 induced by ionizing radiation mediates DNA end-jointing activity, but not apoptosis of thryroid cells. Oncogene 1997 14 1511–1519. (10.1038/sj.onc.1200979) 9129141

[153] Battista S, Martelli ML, Fedele M, et al. A mutated p53 gene alters thyroid cell differentiation. Oncogene 1995 11 2029–2037. 7478522

[154] La Perle KMD, Jhiang SM & Capen CC. Loss of p53 promotes anaplasia and local invasion in ret/PTC1-induced thyroid carcinomas. Am J Pathol 2000 157 671–677. (10.1016/s0002-9440(10)64577-4) 10934169 PMC1850128

[155] McFadden DG, Vernon A, Santiago PM, et al. p53 constrains progression to anaplastic thyroid carcinoma in a Braf-mutant mouse model of papillary thyroid cancer. Proc Natl Acad Sci U S A. 2014 111 E1600–E1609. (10.1073/pnas.1404357111) 24711431 PMC4000830

[156] Zou M, Baitei EY, Al-Rijjal RA, et al. TSH overcomes Braf(V600E)-induced senescence to promote tumor progression via downregulation of p53 expression in papillary thyroid cancer. Oncogene 2016 35 1909–1918. (10.1038/onc.2015.253) 26477313 PMC6310059

[157] Knauf JA, Luckett KA, Chen KY, et al. Hgf/met activation mediates resistance to BRAF inhibition in murine anaplastic thyroid cancers. J Clin Investig 2018 128 4086–4097. (10.1172/jci120966) 29990309 PMC6118575

[158] Untch BR, Dos Anjos V, Garcia-Rendueles MER, et al. Tipifarnib inhibits HRAS-driven dedifferentiated thyroid cancers. Cancer Res 2018 78 4642–4657. (10.1158/0008-5472.can-17-1925) 29760048 PMC6095730

[159] Champa D, Russo MA, Liao XH, et al. Obatoclax overcomes resistance to cell death in aggressive thyroid carcinomas by countering Bcl2a1 and Mcl1 overexpression. Endocr Relat Cancer 2014 21 755–767. (10.1530/erc-14-0268) 25012986 PMC4152557

[160] Nikitski AV, Rominski SL, Condello V, et al. Mouse model of thyroid cancer progression and dedifferentiation driven by STRN-ALK expression and loss of p53: evidence for the existence of two types of poorly differentiated carcinoma. Thyroid 2019 29 1425–1437. (10.1089/thy.2019.0284) 31298630 PMC6797076

[161] Martínez P & Blasco MA. Telomeric and extra-telomeric roles for telomerase and the telomere-binding proteins. Nat Rev Cancer 2011 11 161–176. (10.1038/nrc3025) 21346783

[162] Armanios M & Blackburn EH. The telomere syndromes. Nat Rev Genet 2012 13 693–704. (10.1038/nrg3246) 22965356 PMC3548426

[163] Bernardes de Jesus B & Blasco MA. Telomerase at the intersection of cancer and aging. Trends Genet 2013 29 513–520. (10.1016/j.tig.2013.06.007) 23876621 PMC3896987

[164] Killela PJ, Reitman ZJ, Jiao Y, et al. TERT promoter mutations occur frequently in gliomas and a subset of tumors derived from cells with low rates of self-renewal. Proc Natl Acad Sci U S A 2013 110 6021–6026. (10.1073/pnas.1303607110) 23530248 PMC3625331

[165] Horn S, Figl A, Rachakonda PS, et al. TERT promoter mutations in familial and sporadic melanoma. Science 2013 339 959–961. (10.1126/science.1230062) 23348503

[166] Huang FW, Hodis E, Xu MJ, et al. Highly recurrent TERT promoter mutations in human melanoma. Science 2013 339 957–959. (10.1126/science.1229259) 23348506 PMC4423787

[167] Rheinbay E, Nielsen MM, Abascal F, et al. Analyses of non-coding somatic drivers in 2,658 cancer whole genomes. Nature 2020 578 102–111. (10.1038/s41586-020-1965-x) 32025015 PMC7054214

[168] Liu X, Bishop J, Shan Y, et al. Highly prevalent TERT promoter mutations in aggressive thyroid cancers. Endocr Relat Cancer 2013 20 603–610. (10.1530/erc-13-0210) 23766237 PMC3782569

[169] Vinagre J, Almeida A, Pópulo H, et al. Frequency of TERT promoter mutations in human cancers. Nat Commun 2013 4 2185. (10.1038/ncomms3185) 23887589

[170] Melo M, Da Rocha AG, Vinagre J, et al. TERT promoter mutations are a major indicator of poor outcome in differentiated thyroid carcinomas. J Clin Endocrinol Metab 2014 99 E754–E765. (10.1210/jc.2013-3734) 24476079 PMC4191548

[171] Maloberti T, Repaci A, Poppi L, et al. Exploring the role of TERT in thyroid cancer: a systematic review. Crit Rev Oncol Hematol 2025 213 104792. (10.1016/j.critrevonc.2025.104792) 40482736

[172] Park H, Shin HC, Yang H, et al. Molecular classification of follicular thyroid carcinoma based on TERT promoter mutations. Mod Pathol 2022 35 186–192. (10.1038/s41379-021-00907-6) 34497362 PMC8786663

[173] Mittal P & Roberts CWM. The SWI/SNF complex in cancer – biology, biomarkers and therapy. Nat Rev Clin Oncol 2020 17 435–448. (10.1038/s41571-020-0357-3) 32303701 PMC8723792

[174] Sun X, Chuang JC, Kanchwala M, et al. Suppression of the SWI/SNF component Arid1a promotes Mammalian regeneration. Cell Stem Cell 2016 18 456–466. (10.1016/j.stem.2016.03.001) 27044474 PMC4826298

[175] Dutta A, Sardiu M, Gogol M, et al. Composition and function of mutant Swi/Snf complexes. Cell Rep 2017 18 2124–2134. (10.1016/j.celrep.2017.01.058) 28249159 PMC5837817

[176] Montero-Conde C, Leandro-Garcia LJ, Chen X, et al. Transposon mutagenesis identifies chromatin modifiers cooperating with Ras in thyroid tumorigenesis and detects ATXN7 as a cancer gene. Proc Natl Acad Sci U S A 2017 114 E4951–E4960. (10.1073/pnas.1702723114) 28584132 PMC5488945

[177] Saqcena M, Leandro-Garcia LJ, Maag JLV, et al. SWI/SNF complex mutations promote thyroid tumor progression and insensitivity to redifferentiation therapies. Cancer Discov 2021 11 1158–1175. (10.1158/2159-8290.cd-20-0735) 33318036 PMC8102308

[178] Tchekmedyian V, Dunn L, Sherman E, et al. Enhancing radioiodine incorporation in BRAF-Mutant, radioiodine-refractory thyroid cancers with vemurafenib and the Anti-ErbB3 monoclonal antibody CDX-3379: results of a pilot clinical trial. Thyroid 2022 32 273–282. (10.1089/thy.2021.0565) 35045748 PMC9206492

[179] Liu-Chittenden Y, Huang B, Shim JS, et al. Genetic and pharmacological disruption of the TEAD-YAP complex suppresses the oncogenic activity of YAP. Genes Dev 2012 26 1300–1305. (10.1101/gad.192856.112) 22677547 PMC3387657

[180] Garcia-Rendueles MER, Ricarte-Filho JC, Untch BR, et al. NF2 loss promotes oncogenic RAS-induced thyroid cancers via YAP-dependent transactivation of RAS proteins and sensitizes them to MEK inhibition. Cancer Discov 2015 5 1178–1193. (10.1158/2159-8290.cd-15-0330) 26359368 PMC4642441

[181] Ibrahimpasic T, Xu B, Landa I, et al. Genomic alterations in fatal forms of non-anaplastic thyroid cancer: identification of MED12 and RBM10 as novel thyroid cancer genes associated with tumor virulence. Clin Cancer Res 2017 23 5970–5980. (10.1158/1078-0432.ccr-17-1183) 28634282 PMC5626586

